# Construction of a Comprehensive Contribution Ranking Model for Baijiu Aroma Compounds Based on Multi-Source Molecular Feature Fusion and Validation by Aroma Recombination

**DOI:** 10.3390/foods15173043

**Published:** 2026-08-28

**Authors:** Yashuai Wu, Xudong Zhang, Wenjing Tian, Dongrui Zhao, Mingtao Huang, Jinyuan Sun, Mingquan Huang, Baoguo Sun

**Affiliations:** 1 School of Food Science and Engineering, South China University of Technology, Guangzhou 510640, China; wyss995418706@163.com (Y.W.); huangmt@scut.edu.cn (M.H.); 2 Department of Food and Bioengineering, Beijing Vocational College of Agriculture, Beijing 102442, China; 15027840548@163.com; 3 Key Laboratory of Geriatric Nutrition and Health, Ministry of Education, Beijing Technology and Business University, Beijing 100048, China; sunjinyuan@btbu.edu.cn (J.S.); huangmq@th.btbu.edu.cn (M.H.); sunbg@btbu.edu.cn (B.S.); 4 Key Laboratory of Brewing Molecular Engineering of China Light Industry, Beijing Technology and Business University, Beijing 100048, China; 5 National Baijiu and Huangjiu Research Institute, Beijing Technology and Business University, Beijing 100048, China; 6 China Food Flavor and Nutrition Health Innovation Center, Beijing Technology and Business University, Beijing 100048, China

**Keywords:** Baijiu, aroma compounds, multi-source molecular features, comprehensive contribution ranking, aroma recombination, sensory evaluation

## Abstract

Baijiu aroma arises from the combined effects of diverse volatile and semivolatile trace components within the Baijiu matrix. A single concentration value or odor activity value (OAV) cannot fully characterize the relative importance of a compound in a specific sample. A knowledge base containing 5572 Baijiu aroma-related compounds was used to develop a comprehensive contribution ranking model and a sample-level intensity prediction model. Odor evidence, molecular structure, physicochemical properties, volatility and partitioning characteristics, matrix information, and evidence quality were integrated. Comprehensive contribution is defined here as a within-sample relative ranking index obtained from multi-source molecular features, concentration information, and matrix conditions under a specified model setting. It is not a direct measurement of the true sensory contribution of a compound. After data standardization and screening for feature computability, 5568 compounds were included in the scorer. The R^2^, RMSE, and MAE obtained from 9000 records in the formal independent test set were 0.952018, 0.045684, and 0.036464, respectively. Light-aroma, strong-aroma, and sauce-aroma Baijiu were then analyzed by GC×GC–TOFMS. A total of 732 trace components were obtained, of which 191, 474, and 510 were detected in the three sample groups, respectively. The aroma-related compounds in each sample group were ranked within the group. The top 20 compounds were selected and recombined at their semiquantitative concentrations in the original Baijiu. Sensory evaluation by 10 assessors showed overall similarity scores of 8.70 ± 1.16 and 9.20 ± 1.03 for the light-aroma and strong-aroma recombination samples, respectively. The correlation coefficients between their 10-dimensional sensory profiles and the mean profiles of the original samples were 0.988 and 0.992. The overall similarity score of the sauce-aroma recombination sample was 5.40 ± 1.26. Significant proportional deviations were observed among acidic aroma, sauce-like and roasted aroma, and floral and fruity aroma (*p* < 0.05). These results indicate that multi-source feature fusion can provide an interpretable basis for the relative screening and experimental prioritization of candidate aroma compounds in Baijiu. The rankings for the light-aroma and strong-aroma samples received strong sensory support. However, they should not be interpreted as absolute contributions across aroma types. Evaluation of sauce-aroma Baijiu requires further incorporation of aroma-type-specific prior information, low-OAV components, component interactions, and effects of the authentic Baijiu matrix.

## 1. Introduction

Baijiu is a clear distilled spirit produced from cereal grains through spontaneous mixed culture fermentation. Sorghum is the principal starch source in most traditional processes. Wheat, barley, peas, rice, glutinous rice, and maize may also be used as brewing materials or as substrates for Jiuqu production. Jiuqu supplies a complex community of molds, yeasts, and bacteria. It also provides the enzymes required for starch degradation. Saccharification and alcoholic fermentation therefore occur concurrently within a moist solid grain matrix. The fermented grains are subsequently distilled in the solid state. The fresh distillates are matured and blended before bottling. These operations take place in an open or partly controlled production environment. Microorganisms from Jiuqu, fermentation vessels, tools, workshop surfaces, and the surrounding air can all enter the fermentation system [1,2]. The fermentation vessel is therefore not merely a container. It also functions as an ecological reservoir. Its resident microbiota can alter microbial succession and redirect the formation of acids, alcohols, esters, aldehydes, ketones, and other aroma compounds. The final aroma type is consequently shaped by the combined effects of grain composition, starter preparation, fermentation temperature, oxygen exposure, vessel ecology, and process recycling.

The three aroma types examined in this study arise from different configurations of this shared production framework. Representative light-aroma Baijiu is commonly produced from sorghum with low-temperature Daqu prepared from barley and peas. Alcoholic fermentation is usually conducted in ceramic jars rather than continuously used mud pits. This environment limits the persistent influence of pit mud microbiota and contributes to a clean, mild, and relatively delicate aroma profile. The jar surface and workshop air remain important microbial sources. Changes in these environmental communities can affect ester formation and cause variations among production periods [2]. Strong-aroma Baijiu may be produced from sorghum alone or from mixtures containing sorghum, wheat, rice, glutinous rice, and maize. Medium- or medium-high-temperature Daqu is generally used. Fermentation proceeds in mud-lined pits that are repeatedly used over many production cycles. Mature pit mud harbors anaerobic microbial consortia involved in the generation of short-chain fatty acids and their corresponding ethyl esters. The reuse of pit mud and the recycling of fermented grains gradually reinforce the rich fruity aroma and characteristic cellar notes of this style [1,3]. Sauce-aroma Baijiu mainly uses sorghum as its starch source and wheat-based high-temperature Daqu as the starter. Its production alternates open stacking fermentation with sealed pit fermentation over multiple rounds. A representative traditional cycle lasts approximately 1 year and includes two grain feeding operations, eight fermentation rounds, and seven rounds of base liquor collection. Distillates obtained from different rounds are aged separately and later blended. This process imposes strong thermal selection and permits repeated environmental inoculation. It also produces substantial spatial and temporal heterogeneity within the fermentation system [1,4]. Sauce, roasted, nutty, fruity, and fermented notes therefore emerge from a broader and more interactive metabolite network. These technological contrasts provide a biological basis for the differences in volatile composition and sensory expression among the three Baijiu styles. They also help explain why a limited set of volatile compounds may reproduce one aroma type more successfully than another.

Baijiu aroma is not a simple superposition of a few abundant substances. It reflects the combined expression of volatile and semivolatile trace components in the Baijiu matrix, including esters, alcohols, acids, aldehydes, ketones, sulfur-containing compounds, nitrogen-containing compounds, and aromatic compounds [5,6,7,8,9]. Concentrations and sensory thresholds vary substantially among compounds. Some compounds at low concentrations produce distinctive odors because of their low olfactory thresholds. In contrast, the effects of some abundant compounds may be limited by volatility, gas–liquid partitioning, and the Baijiu matrix. Synergistic, masking, counteractive, and modifying effects can also occur in mixtures. Therefore, the odor characteristics of an individual compound cannot be equated directly with its function in an authentic Baijiu matrix. Baijiu aroma research must identify not only which compounds are present but also which candidates should be prioritized for validation in a specific sample.

Key aroma compounds are commonly screened using MS-based chromatography, GC-O, OAV, aroma recombination, and omission tests [10,11,12,13,14,15,16]. These methods have established the basic framework for Baijiu aroma analysis, but each has limitations. OAV is calculated from the ratio of concentration to threshold and is easy to interpret. However, it depends strongly on the threshold medium, assessor population, analytical conditions, and data units. Thresholds measured in water, air, hydroalcoholic solutions, and authentic Baijiu matrices are not identical. GC-O can identify odor-active chromatographic peaks but does not fully represent compound release from liquid Baijiu. Aroma recombination and omission tests are closer to sensory reality, but they are costly and often rely on prior experience when candidates are selected. Experimental validation of every compound in a large candidate set is impractical.

These limitations indicate that candidate aroma compounds cannot be prioritized using concentration, threshold, or a single structural index alone. Molecular structure and functional groups affect odor properties and molecular recognition [17,18,19,20,21,22]. Molecular weight, polarity, hydrophobicity, hydrogen-bonding capacity, and topology influence solubility, migration, and partitioning. Boiling point, vapor pressure, Henry’s law constant, and gas–liquid partitioning affect release from the liquid into the headspace. Alcohol content, pH, temperature, and storage conditions further modify these properties in a specific sample [23,24,25]. Integrating structural, physicochemical, energetic, volatility, odor, and Baijiu-occurrence evidence enable candidate compounds to be compared from multiple perspectives.

Accordingly, a comprehensive contribution ranking model based on multi-source molecular feature fusion was developed using a knowledge base of 5572 Baijiu aroma-related compounds. The knowledge base contains compound identifiers, molecular descriptors, molecular fingerprints, force-field energies, quantum chemical parameters, volatility and partitioning properties, chemical classes, odor descriptors, sensory thresholds, and evidence of occurrence in Baijiu. Data quality, evidence level, and scope of applicability were incorporated because the completeness and strength of evidence differed among data sources. This design allows each ranking to be interpreted in relation to its information basis [26,27,28]. Comprehensive contribution is defined as a within-sample relative ranking index generated under a specified model setting. It integrates odor evidence, molecular structure, physicochemical properties, volatility and partitioning, measured concentration, the Baijiu matrix, and evidence quality. The index does not represent the absolute sensory contribution of a compound and should not be compared directly among aroma types or samples. It is intended to identify aroma-related compounds that deserve priority in experimental validation and to explain the main basis for their rankings [29,30,31,32,33,34,35]. The top 20 compounds in each target original Baijiu were subsequently recombined at their semiquantitative concentrations. Their major aroma dimensions, overall intensity, harmony, and similarity were compared with those of the original Baijiu [5,32,33,34,35].

In this study, comprehensive contribution is an operational term for relative model-based prioritization. It indicates the position of a compound relative to other compounds in the same sample under the specified model settings. A higher score therefore means a higher priority for subsequent experimental evaluation. It does not indicate a quantitatively greater sensory effect. The score is dimensionless and cannot be interpreted as the percentage of aroma perceived by assessors. The normalized contribution proportion represents only the share of the summed model scores within a sample. It is not a sensory contribution percentage. Unless an experimentally observed sensory effect is explicitly stated, all subsequent uses of contribution, contribution score, and contribution proportion refer to these relative ranking quantities.

This study aimed to establish an analytical workflow that connects large-scale compound information with actual aroma performance. The workflow comprises knowledge-based curation, multi-source feature fusion, within-sample relative ranking, and validation by aroma recombination. It is not intended to replace quantitative analysis, OAV evaluation, recombination, or omission tests. Instead, it provides an interpretable basis for initial candidate screening and experimental prioritization. It also offers a technical framework for subsequent Baijiu characterization and targeted validation.

## 2. Materials and Methods

### 2.1. Baijiu Samples

Representative samples of three Baijiu aroma types were selected and coded A-1, A-2, A-3, B-1, B-2, B-3, C-1, C-2, and C-3. Group A represented light-aroma Baijiu and had an alcohol content of 53% vol and a pH of 4.624. Group B represented strong-aroma Baijiu and had an alcohol content of 52% vol and a pH of 4.163. Group C represented sauce-aroma Baijiu and had an alcohol content of 53% vol and a pH of 3.638. Sample codes were used throughout the main text to prevent brand and producer information from affecting the objective interpretation of the results (*n* = 3). Group A consisted of a commercial Fenjiu product manufactured by Shanxi Xinghuacun Fen Wine Factory Co., Ltd. in Fenyang, Shanxi Province, China. Group B consisted of a commercial Wuliangye product manufactured by Wuliangye Yibin Co., Ltd. in Yibin, Sichuan Province, China. Group C consisted of a commercial Xijiu product manufactured by Guizhou Xijiu Co., Ltd. in Xijiu Town, Xishui County, Guizhou Province, China. All 3 products were sampled in 2025. A single production batch was represented for each aroma style. Sample selection was based on the declared aroma type and the use of established commercial products as sample-specific test cases. The numerical suffixes 1, 2, and 3 indicate 3 replicate analytical preparations of the same commercial sample. They do not represent 3 independent products or 3 independent production batches. The value *n* = 3 therefore refers to analytical replication within each group. This sampling design allowed the analytical workflow and aroma recombination strategy to be examined across 3 contrasting Baijiu aroma styles. It was not designed to quantify variation among brands, producers, geographical regions, production batches, aging periods, or storage histories. The compositional and sensory differences reported in this study should therefore be interpreted as differences among the 3 selected products rather than universal differences among all Baijiu products belonging to these aroma styles.

### 2.2. Compound Standards

Pentyl acetate, 4-octanol, and 2-ethylbutanoic acid were used as internal standards for semiquantitative analysis. Pentyl acetate had CAS No. 628-63-7 and an internal-standard solution concentration of 2500.00 mg/L; 4-octanol had CAS No. 589-62-8 and a concentration of 250.00 mg/L; 2-ethylbutanoic acid had CAS No. 88-09-5 and a concentration of 1000.00 mg/L (Beijing InnoChem Science and Technology Co., Ltd., Beijing, China). Pentyl acetate, 4-octanol, and 2-ethylbutanoic acid were used for the semiquantitative analysis of esters, alcohols, and acids, respectively. For other chemical classes, the internal standard with the greatest molecular structural similarity was selected from these three standards. This procedure was used to reduce structural and instrumental response differences between each internal standard and the corresponding analyte.

The following standards were used in the Baijiu recombination experiment. Ester standards included ethyl acetate, ethyl 2-methylbutanoate, ethyl 3-methylbutanoate, ethyl pentanoate, ethyl hexanoate, ethyl heptanoate, ethyl octanoate, ethyl decanoate, ethyl dodecanoate, ethyl lactate, diethyl succinate, ethyl levulinate, hexyl acetate, butyl hexanoate, and hexyl hexanoate. Acid standards included acetic acid, propanoic acid, 2-methylpropanoic acid, butanoic acid, 3-methylbutanoic acid, pentanoic acid, hexanoic acid, heptanoic acid, and octanoic acid. Alcohol standards included 1-butanol and 3-methyl-1-butanol. Standards from other chemical classes included acetoin, 1,1-diethoxy-3-methylbutane, and D-mannosamine. All compounds were of chromatographic purity or analytical-standard grade and were purchased from Shanghai Macklin Biochemical Technology Co., Ltd., Shanghai, China.

Dichloromethane and absolute ethanol used for liquid–liquid extraction and sample preparation were of chromatographic grade and were purchased from Shanghai Aladdin Biochemical Technology Co., Ltd., Shanghai, China. Sodium chloride was of analytical grade and was purchased from J&K Scientific Ltd., Beijing, China. Anhydrous sodium sulfate was of analytical grade and was purchased from Sinopharm Chemical Reagent Co., Ltd., Shanghai, China. Ultrapure water was produced using an ultrapure water system.

### 2.3. Instruments

A Pegasus 4D GC×GC–TOFMS system (LECO Corporation, St. Joseph, MI, USA) was equipped with a 7890B GC (Agilent Technologies Inc., Santa Clara, CA, USA), an independent secondary oven (LECO Corporation, St. Joseph, MI, USA), a QuadJet™ four-jet liquid-nitrogen cryogenic thermal modulator (LECO Corporation, St. Joseph, MI, USA), and a time-of-flight mass detector (LECO Corporation, St. Joseph, MI, USA). Instrument control, data acquisition, chromatographic peak extraction, mass spectral deconvolution, and library searching were performed using ChromaTOF^®^ software (version 4.72, LECO Corporation, St. Joseph, MI, USA). The first-dimension column was an Agilent J&W DB-WAX fused-silica capillary column (30 m × 0.25 mm × 0.25 μm, Agilent Technologies Inc., Santa Clara, CA, USA). The second-dimension column was an Agilent J&W DB-5 fused-silica capillary column (2 m × 0.25 mm × 0.10 μm, Agilent Technologies Inc., Santa Clara, CA, USA). Additional instruments included a GGC-C vertical separatory-funnel shaker (Beijing Guohuan Gaoke Automation Technology Research Institute, Beijing, China), an N-EVAP 111 nitrogen evaporator (Organomation Associates Inc., West Berlin, MA, USA), and a GenPure UV-TOC xCAD Plus ultrapure water system (Thermo Fisher Scientific Inc., Waltham, MA, USA). An ME204E analytical balance with a maximum capacity of 220 g and a readability of 0.1 mg (Mettler-Toledo GmbH, Greifensee, Switzerland) and a SevenCompact S220 benchtop pH meter (Mettler-Toledo GmbH, Greifensee, Switzerland) were also used.

### 2.4. Experimental Methods

#### 2.4.1. Liquid–Liquid Extraction of Baijiu Aroma-Related Components

A 25.0 mL aliquot of each Baijiu sample was accurately measured. The alcohol content was adjusted to 15% vol with saturated sodium chloride solution prepared in ultrapure water to increase ionic strength and promote partitioning of aroma-related compounds into the organic phase [36]. Each sample was supplemented with 1 mL of the mixed internal-standard working solution. The added amounts of pentyl acetate, 4-octanol, and 2-ethylbutanoic acid were kept constant. Dichloromethane (50 mL) was then added, and the sample was thoroughly extracted using a vertical separatory-funnel shaker. After phase separation, the lower organic phase was collected. The extraction was repeated three times, and the organic phases were combined. Anhydrous sodium sulfate was added until the mixture flowed freely to ensure complete water removal. The mixture was held overnight at −4 °C. After filtration, the organic phase was initially concentrated using a Vigreux distillation apparatus. When the extract volume reached approximately 1.0 mL, it was transferred to a nitrogen evaporation tube and further concentrated to 1.0 mL under a gentle nitrogen stream. The concentrate was transferred to a sealed chromatographic vial for GC×GC–TOFMS analysis. Each Baijiu sample was extracted independently in triplicate [37,38,39,40].

#### 2.4.2. GC×GC–TOFMS Conditions

Volatile and semivolatile aroma-related components in the Baijiu samples were analyzed using a Pegasus 4D GC×GC–TOFMS system (LECO Corporation, St. Joseph, MI, USA). An Agilent J&W DB-WAX fused-silica capillary column (30 m × 0.25 mm × 0.25 μm, Agilent Technologies Inc., Santa Clara, CA, USA) was used in the first dimension. An Agilent J&W DB-5 fused-silica capillary column (2 m × 0.25 mm × 0.10 μm, Agilent Technologies Inc., Santa Clara, CA, USA) was used in the second dimension. The columns were connected in series. The initial temperature of the first oven was 50 °C and was held for 2 min. It was increased to 150 °C at 3 °C/min and then to 230 °C at 5 °C/min, followed by a 10 min hold. The second oven was maintained 5 °C above the first oven. A QuadJet™ dual-stage cryogenic thermal modulator (LECO Corporation, St. Joseph, MI, USA) was used with liquid nitrogen as the coolant. The modulator was maintained 15 °C above the first oven. The modulation period was 5 s, with a hot-jet time of 1.0 s and a cold-jet time of 1.5 s. High-purity gaseous nitrogen generated by vaporization and pressure reduction of liquid nitrogen was used as the carrier gas. Its purity was at least 99.999%. A constant flow of 1.0 mL/min was applied. The inlet temperature was 250 °C, and 1.0 μL was injected in splitless mode. The time-of-flight MS was operated in electron-impact ionization mode at 70 eV. The ion-source and transfer-line temperatures were 230 and 250 °C, respectively. The mass range was *m*/*z* 35 to 350, and the acquisition rate was 100 spectra/s. Instrument operation, raw-data acquisition, and preliminary processing were performed using ChromaTOF software (version 4.72, LECO Corporation, St. Joseph, MI, USA).

#### 2.4.3. Qualitative Analysis of Compounds

Raw GC×GC–TOFMS data were processed using ChromaTOF^®^ software (LECO Corporation, St. Joseph, MI, USA). Processing included automatic peak detection, baseline correction, mass spectral deconvolution, and two-dimensional peak matching. Spectra were searched against the mass spectral database supplied with the software [36,37,38,39]. Compounds were tentatively identified by matching deconvoluted mass spectra to reference spectra in the database. After manual verification, compound names, first- and second-dimension retention times, and peak areas were exported to construct the data matrix used for subsequent multivariate analysis and model training.

#### 2.4.4. Semiquantitative Analysis Based on Structural Similarity

To reduce quantitative bias among chemical classes when corresponding standards or isotopically labeled internal standards were unavailable, a tiered internal-standard selection strategy was used. Chemical class was prioritized, followed by structural similarity. Pentyl acetate, 4-octanol, and 2-ethylbutanoic acid were assigned as internal standards for esters, alcohols, and carboxylic acids, respectively. For ketones, aldehydes, ethers, heterocyclic compounds, nitrogen-containing compounds, and other classes, the most structurally similar of the three internal standards was selected using two-dimensional molecular fingerprint similarity [26,27].

The standard English names of the compounds were converted to SMILES, and the resulting structures were checked for valence and standardized [41]. Morgan circular fingerprints were generated using RDKit 2026.03.3. The primary fingerprint parameters were a radius of r=2, a fingerprint length of L=2048 bits, inclusion of bond-type information, and exclusion of chirality encoding. A radius of 2 indicates that local topological environments were expanded from each central atom to a maximum of two bonds, which generally corresponds to ECFP4. Morgan/ECFP fingerprints encode the local environments of different central atoms into comparable discrete structural features [26].

To illustrate the circular fingerprint encoding process, query compound x was represented as a molecular graph.

$$G_{x}=\left(V_{x},E_{x}\right)$$

where $V_{x}$ is the set of atoms and $E_{x}$ is the set of chemical bonds. For atom $i\in V_{x}$, the initial atom invariants can be abstractly expressed as follows.

$$u_{i}^{\left(0\right)}=\left(Z_{i},d_{i},v_{i},q_{i},n_{H,i},r_{i}\right)$$

where $Z_{i}$ is atomic number, $d_{i}$ is atomic connectivity, $v_{i}$ is valence state, $q_{i}$ is formal charge, $n_{H,i}$ is the number of attached hydrogen atoms, and $r_{i}$ is the ring-related indicator. The initial identifier was generated using the deterministic hash function $H\left(\cdot \right)$.

$$I_{i}^{\left(0\right)}=H\;\left(u_{i}^{\left(0\right)}\right)$$

The above expression provides a mathematical abstraction of the initial atom invariants used in circular fingerprints. In the actual calculation, the default ECFP-type atom-invariant generator in RDKit was used. At iteration t, the previous identifier of the central atom, the identifiers of adjacent atoms, and the corresponding bond types were jointly encoded.

$$I_{i}^{\left(t\right)}=H\;\left[I_{i}^{\left(t-1\right)},sort\left\{\left(B_{ij},I_{j}^{\left(t-1\right)}\right)|j\in N\left(i\right)\right\}\right],\;t=1,2$$

where $N\left(i\right)$ is the set of atoms adjacent to atom i, $B_{ij}$ is the bond-type code between atoms i and j, and $sort\left(\cdot \right)$ denotes deterministic sorting of neighborhood information before hashing. After iterations at radii 0, 1, and 2, the set of local structural environments for compound x can be expressed as follows.

$$I_{x}=\left\{I_{i}^{\left(t\right)}|i\in V_{x},t\in \{0,1,2\}\right\}$$

Each environment identifier was then folded into a binary fingerprint space with a length of L=2048. For any environment identifier $I_{i}^{\left(t\right)}$, the corresponding fingerprint position was calculated as follows.

$$k_{i,t}=I_{i}^{\left(t\right)}mod\;L$$

Bit k of the binary fingerprint was defined as follows.

$$f_{x,k}=\left\{\begin{array}{ll} 1, & \exists \left(i,t\right),\;k_{i,t}=k\\ 0, & \mathrm{otherwise} \end{array}\right.$$

The Morgan fingerprint vector of compound x was thus obtained as follows.

$$f_{x}=\left(f_{x,1},f_{x,2},\ldots ,f_{x,L}\right),\;L=2048$$

Structural similarity between a query compound and an internal standard was calculated using the Tanimoto coefficient. Let the binary fingerprints of query compound x and candidate internal standard $s_{j}$ be $f_{x}$ and $f_{s_{j}}$, respectively. The numbers of bits set to 1 in the two fingerprints and the number of shared bits set to 1 were calculated as follows.

$$a_{x}=\sum_{k=1}^{L}f_{x,k}$$

$$a_{s_{j}}=\sum_{k=1}^{L}f_{s_{j},k}$$

$$c_{x,s_{j}}=\sum_{k=1}^{L}f_{x,k}f_{s_{j},k}$$

where $a_{x}$ and $a_{s_{j}}$ are the numbers of fingerprint features in the two molecules, and $c_{x,s_{j}}$ is the number of shared fingerprint features. The Tanimoto similarity of the Morgan fingerprints was defined as follows.

$$S_{Morgan}\left(x,s_{j}\right)=\frac{c_{x,s_{j}}}{a_{x}+a_{s_{j}}-c_{x,s_{j}}}$$

An equivalent vector form is given below.

$$S_{Morgan}\left(x,s_{j}\right)=\frac{f_{x}^{T}f_{s_{j}}}{∥f_{x}{∥}_{1}+∥f_{s_{j}}{∥}_{1}-f_{x}^{T}f_{s_{j}}}$$

The coefficient ranges from 0 to 1. A value closer to 1 indicates that the molecules share more two-dimensional local topological features under the selected fingerprint definition. The Tanimoto coefficient is the ratio of the intersection to the union of two binary feature sets and is suitable for molecular fingerprint similarity comparisons [27]. For each compound that was not an ester, alcohol, or carboxylic acid, similarities to pentyl acetate $s_{1}$, 4-octanol $s_{2}$, and 2-ethylbutanoic acid $s_{3}$ were calculated to form the following similarity vector.

$$S_{x}=\left[S_{Morgan}\left(x,s_{1}\right),S_{Morgan}\left(x,s_{2}\right),S_{Morgan}\left(x,s_{3}\right)\right]$$

When the maximum Morgan similarity was unique, the candidate internal standard was selected using the following equation.

$$j_{sel}=\underset{j\in \{1,2,3\}}{arg\;max}\;S_{Morgan}\left(x,s_{j}\right)$$

Ties in the maximum similarity can occur for small molecules, molecules of low complexity, or fingerprints affected by hash folding. A hierarchical decision procedure was therefore established. The candidate set with the maximum Morgan similarity was defined as follows.

$$J_{1}=\left\{j\in \{1,2,3\}|S_{Morgan}\left(x,s_{j}\right)=\underset{l\in \{1,2,3\}}{max}S_{Morgan}\left(x,s_{l}\right)\right\}$$

When $card\left(J_{1}\right)>1$, MACCS fingerprints based on 166 public structural keys were used for secondary comparison [42]. Let the MACCS fingerprints of the query compound and candidate internal standard be $m_{x}$ and $m_{s_{j}}$, respectively. Their Tanimoto similarity was calculated as follows.

$$S_{MACCS}\left(x,s_{j}\right)=\frac{m_{x}^{T}m_{s_{j}}}{∥m_{x}{∥}_{1}+∥m_{s_{j}}{∥}_{1}-m_{x}^{T}m_{s_{j}}}$$

Among the candidates tied by Morgan similarity, the set with the maximum MACCS similarity was defined as follows.

$$J_{2}=\left\{j\in J_{1}|S_{MACCS}\left(x,s_{j}\right)=\underset{l\in J_{1}}{max}S_{MACCS}\left(x,s_{l}\right)\right\}$$

If $card\left(J_{2}\right)=1$, that internal standard was selected. If the Morgan and MACCS similarities remained tied, the absolute difference in relative molecular mass between the query compound and candidate internal standard was used as the third-level criterion.

$$\Delta M_{j}=\left|M_{x}-M_{s_{j}}\right|$$

$$j_{sel}=\underset{j\in J_{2}}{arg\;min}\;\Delta M_{j}$$

After chemical-class information and structural similarity were integrated, $g\left(x\right)\in \{E,L,C,O\}$ represented esters, alcohols, carboxylic acids, and other compounds, respectively. In addition, $s_{1}$, $s_{2}$, and $s_{3}$ represented pentyl acetate, 4-octanol, and 2-ethylbutanoic acid, respectively. The final internal-standard selection rule was expressed as follows.

$$s_{sel}\left(x\right)=\left\{\begin{array}{ll} s_{1}, & g\left(x\right)=E\\ s_{2}, & g\left(x\right)=L\\ s_{3}, & g\left(x\right)=C\\ s_{j_{sel}}, & g\left(x\right)=O \end{array}\right.$$

After the internal standard was selected, analyte concentrations were calculated from peak-area ratios of corresponding replicates. Thus, analyte AREA-1 was paired with internal-standard AREA-1, AREA-2 with AREA-2, and AREA-3 with AREA-3. The response factors of the analyte and internal standard were defined as follows.

$$RF_{x}=\frac{A_{x}}{C_{x}},\;\;RF_{IS}=\frac{A_{IS}}{C_{IS}}$$

The response factor of the analyte relative to the internal standard was defined as follows.

$$RRF_{x,IS}=\frac{RF_{x}}{RF_{IS}}=\frac{A_{x}/C_{x}}{A_{IS}/C_{IS}}$$

Therefore, the concentration of the query compound in replicate i was calculated as follows.

$$C_{x,i}=\frac{A_{x,i}}{A_{IS,i}}\times \frac{C_{IS}}{RRF_{x,IS}},\;i=1,2,3$$

where $A_{x,i}$ is the peak area of the query compound in replicate i, $A_{IS,i}$ is the peak area of the corresponding internal standard in the same replicate, and $C_{IS}$ is the working concentration of the internal standard. Because standard curves and experimental relative response factors were unavailable for all query compounds, $RRF_{x,IS}=1$ was set to 1 for all calculations. The equation was therefore simplified as follows.

$$C_{x,i}=\frac{A_{x,i}}{A_{IS,i}}\times C_{IS}$$

The mean peak area and sample standard deviation for the three replicates were calculated as follows.

$${\bar{A}}_{x}=\frac{1}{3}\sum_{i=1}^{3}A_{x,i}$$

$$s_{A}=\sqrt{\frac{\sum_{i=1}^{3}{\left(A_{x,i}-{\bar{A}}_{x}\right)}^{2}}{3-1}}$$

The corresponding mean concentration and sample standard deviation were calculated as follows.

$${\bar{C}}_{x}=\frac{1}{3}\sum_{i=1}^{3}C_{x,i}$$

$$s_{C}=\sqrt{\frac{\sum_{i=1}^{3}{\left(C_{x,i}-{\bar{C}}_{x}\right)}^{2}}{3-1}}$$

Results were reported as ${\bar{A}}_{x}\pm s_{A}$ and ${\bar{C}}_{x}\pm s_{C}$. Morgan and MACCS fingerprints describe similarities in two-dimensional topology and predefined structural fragments [26,27,41,42]. However, they do not fully represent differences in compound responses during GC injection, chromatographic transfer, electron-impact ionization, fragmentation, and MS detection [36,37,38,39,40].

#### 2.4.5. Sensory Validation of Recombined Baijiu Samples

To evaluate the practical aroma interpretability of the model results, the 20 highest-scoring compounds were selected from sample groups A, B, and C. Each compound was added at its internal-standard-based semiquantitative concentration in the corresponding sample to prepare recombination samples A-A, B-B, and C-C. The recombination matrices were prepared from commercial food-grade ethanol and ultrapure water. Their alcohol contents were adjusted to 53% vol, 52% vol, and 53% vol to match the corresponding original samples. No additional pH adjustment was performed. The selected acids were added at their measured concentrations together with the other flavor compounds. This approach avoided changes in composition caused by exogenous acid or base reagents. Each recombination sample was compared with its corresponding original sample from groups A, B, or C. Three independent replicates were prepared for each sample group.

Sensory evaluation was conducted by 10 assessors from the Sensory Laboratory of Beijing Technology and Business University. The panel included five men and five women with extensive experience in alcoholic beverage evaluation. A 15 mL aliquot was placed in a standard Baijiu tasting glass, covered, and equilibrated at 20 ± 1 °C. Samples were labeled with random three-digit codes and presented in random order. Before formal evaluation, terminology and scale use were standardized through training based on the characteristic aromas of light-aroma, strong-aroma, and sauce-aroma Baijiu. This design supported assessor consistency and comparability among aroma types. The same 10 assessors evaluated all 9 original sample preparations and all 3 recombination samples. The assessor was therefore the repeated experimental unit. This arrangement formed a complete within-assessor repeated measures design. Panel training was conducted with representative samples of the 3 Baijiu aroma styles. It covered recognition of the 8 aroma attributes, interpretation of the definitions in Table 1, differentiation between closely related descriptors, and consistent use of the 0 to 10 scale. Repeated assessments were included during training to improve individual repeatability and agreement within the panel. Formal evaluation began only after the assessors had established a common understanding of the descriptors and scale anchors. Consensus discussion was permitted during training but not during formal scoring. These procedures followed ISO 8586:2023 [43], ISO 11132:2021 [44], and ISO 13299:2016 [45]. The evaluations were conducted in individual sensory booths under controlled conditions consistent with ISO 8589:2007 [46]. Samples were presented one at a time in identical covered glasses. Separate random presentation sequences were prepared for individual assessors. This procedure distributed potential order and carryover effects across the panel. For aroma profile evaluation, assessors were blinded to brand identity, sample composition, compound ranking, and sample code meaning. For overall similarity evaluation, the corresponding original Baijiu was provided as a coded reference and the recombination sample was provided with a different code. Assessors were informed only that similarity should be judged against the coded reference. They received no information about the selected compounds or expected reconstruction performance. All scores were recorded independently. The sample identities and code key were not disclosed until all evaluations had been completed.

Sensory evaluation was restricted to the orthonasal aroma of the Baijiu samples. The evaluated dimensions were fruity and estery aroma, floral aroma, grain aroma, pit and fermentation aroma, sauce-like and roasted aroma, acidic aroma, alcoholic aroma, and sweet aroma. Overall aroma intensity, aroma harmony, and overall similarity between each recombination sample and its corresponding original sample were also evaluated. All attributes were rated on a scale from 0 to 10. Detailed definitions are provided in Table 1.

#### 2.4.6. Data Analysis and Modeling Software

All experiments were conducted in triplicate, and results are expressed as the mean ± standard deviation. R software (version 4.4.2; R Foundation for Statistical Computing, Vienna, Austria) was used for data organization, one-way ANOVA, Tukey’s multiple comparisons, and visualization. Statistical significance was defined as *p* < 0.05.

Sensory data were analyzed separately according to the within-assessor structure. The assessor was used as the statistical unit. Overall similarity scores for AA, BB, and CC were compared using a one-factor repeated measures ANOVA. The recombination model was treated as the within-assessor factor. Sphericity was evaluated using the Mauchly test. A Greenhouse Geisser correction was planned when the sphericity assumption was not satisfied. Significant omnibus effects were examined using paired comparisons with Holm adjustment. For each aroma style, the 3 evaluations of the original sample were averaged within each assessor. This assessor-specific mean was compared with the score assigned by the same assessor to the corresponding recombination sample. Paired *t*-tests were performed for the 10 sensory dimensions. Holm adjustment was applied separately within each aroma style to control the error arising from multiple comparisons. Overall similarity results were reported as the mean, standard deviation, and 95% confidence interval. Reconstruction effects for individual attributes were expressed as mean paired differences with 95% confidence intervals.

PCA and OPLS-DA were performed using SIMCA 14.1 to compare overall differences in flavor components among sample groups A, B, and C. Compounds that contributed strongly to sample classification were screened using VIP values. Variable importance in projection, abbreviated as VIP, provides a dimensionless measure of the weighted contribution of each original X variable to the latent components used by PLS-DA to explain class membership. Its calculation combines the squared weight of a variable on each retained component with the amount of Y variance explained by that component [47,48]. A larger VIP therefore indicates that the variable has a stronger influence on the fitted separation among the 3 Baijiu groups. A smaller value indicates a weaker weighted contribution within the latent variable structure. VIP scores are nonnegative and are not restricted to the interval between 0 and 1. Under standard normalization, the mean of the squared VIP scores across all *p*-modeled variables equals 1. The theoretical range is consequently from 0 to the square root of *p*. A value above 1 is commonly regarded as evidence of above-average influence. Values between 0.8 and 1 may retain moderate discriminatory information. Values below 0.8 are generally considered weak [48,49]. These boundaries are practical screening rules rather than formal tests of statistical significance. In the present study, VIP greater than 1 was used to identify potential differential trace components. A high VIP was interpreted only as strong relevance to discrimination among groups A, B, and C. It was not treated as evidence of high concentration, a large odor activity value, or a direct sensory contribution. Interpretation also considered the concentration pattern, within-group variation, chemical class, and correlation with other compounds.

R was used for statistical calculations and plotting, whereas SIMCA supported multivariate analyses such as PCA and OPLS-DA. The comprehensive contribution ranking model for Baijiu aroma compounds was implemented and executed in Python 3.11.13. The sample-level calibration was fitted using the deterministic ridge regression procedure described in Supplementary S5. RDKit 2026.03.3 was used consistently for SMILES validation and standardization, Morgan fingerprint generation, molecular descriptor calculation, and MMFF94 energy calculation. The same RDKit version was applied throughout the final workflow. Structural features produced with different RDKit releases were not combined in the released dataset or scoring model. Molecular energies were calculated using OpenMM 8.5.2 and the Sage 2.2.1 force field. Quantum chemical calculations were performed using Psi4 1.11. Volatility, solubility, and partitioning properties were predicted using OPERA 2.9.2.

#### 2.4.7. Comparison with Simpler Ranking Approaches

The comprehensive score ranking was compared with 2 simpler reference approaches within each sample group. The concentration-based ranking was obtained by sorting all scored compounds in descending order of their mean semiquantitative concentrations. The intended OAV-based ranking was defined using OAV_i_ = C_i_ divided by T_i_, where C_i_ is the concentration of compound i and T_i_ is its odor threshold. Numerical compound-specific thresholds were not retained in the released scoring artifact. The established missing threshold rule therefore assigned T_i_ a common prior value of 1.0 mg/L. Under this condition, the prior-based OAV ranking is mathematically identical to the concentration ranking. It was retained as a sensitivity reference that shows the consequence of missing threshold information. It was not treated as a conventional OAV analysis based on experimentally determined compound-specific thresholds.

Agreement between the comprehensive score and each reference ranking was evaluated using Spearman rank correlation. Agreement among the highest-priority candidates was evaluated by counting the compounds shared between the corresponding top 20 lists. All comparisons were performed separately for light-aroma, strong-aroma, and sauce-aroma Baijiu. This design examines whether the comprehensive score merely reproduces the abundance order or changes the prioritization after odor evidence, molecular structure, physicochemical properties, matrix adjustment, and evidence quality are incorporated. Differences among ranking methods were interpreted as changes in prioritization rather than proof that one method was sensorially superior. A complete OAV benchmark will require harmonized numerical thresholds determined in hydroalcoholic media that closely match the alcohol content and composition of the corresponding Baijiu sample. This requirement is important because ethanol content and interactions among aroma compounds can alter olfactory thresholds and mixture perception [17,18,50,51].

## 3. Results and Analysis

### 3.1. Differences in Trace Component Profiles Among Three Baijiu Aroma Types and Screening of Potential Markers

Light-aroma, strong-aroma, and sauce-aroma Baijiu samples were compared using trace-component concentrations and molecular structural information. The three sample groups were designated A, B, and C, respectively, with three replicate measurements per group. After compound-name standardization, consolidation of duplicate results, and correction of anomalous data, 732 trace components were obtained. Their concentrations and structural parameters are provided in Supplementary S1. Groups A, B, and C contained 191, 474, and 510 detected compounds, corresponding to detection rates of 26.1%, 64.8%, and 69.7%, respectively. Sauce-aroma Baijiu had the broadest compound coverage, with 36 more compounds than strong-aroma Baijiu and 319 more than light-aroma Baijiu. Thus, the sauce-aroma and strong-aroma samples in this batch had broader trace-component profiles, whereas the light-aroma profile was more concentrated.

A total of 121 compounds were shared by all three sample groups, accounting for 16.5% of all compounds. Groups A, B, and C contained 37, 168, and 205 unique components, respectively. Groups B and C shared 168 compounds that were not detected in group A. In contrast, only 17 and 16 compounds were shared exclusively by A and B and by A and C, respectively. Compound coverage was therefore more similar between strong-aroma and sauce-aroma Baijiu, whereas light-aroma Baijiu showed a more distinct profile. These differences may be related to starter-production temperature, fermentation duration, the fermentation-pit environment, and microbial metabolic pathways [52,53,54]. Previous studies have shown that the style of each Baijiu aroma type is not determined by a single compound. Instead, it results from the combined effects of esters, acids, alcohols, aldehydes, nitrogen-containing compounds, sulfur-containing compounds, and other component classes [5,6,7,8,9]. More than 2700 trace compounds have been reported in Baijiu. Key differences among aroma types are mainly reflected in component combinations and proportions rather than simple presence or absence [5].

The sums of the mean trace-component concentrations in groups A, B, and C were 31,260.051, 32,009.250, and 63,781.330 mg/L, respectively. The total for group C was 2.040 times that of A and 1.993 times that of B, whereas A and B differed by only 2.4%. These totals are mathematical sums of semiquantitative concentrations. They can be affected by highly responsive substances, extreme values, and tentative identifications. They are suitable for describing the overall abundance of this dataset but should not be equated directly with true flavor intensity. The cumulative acid and ester concentrations in group C were 17,813.281 and 17,795.511 mg/L, respectively, and dominated its concentration profile. In group B, the total ester concentration was 10,230.693 mg/L, exceeding the acid concentration of 5994.100 mg/L. In group A, the total ester, acid, and alcohol concentrations were 4344.387, 2826.748, and 1930.095 mg/L, respectively. Esters generally form the principal aroma framework of strong-aroma Baijiu. Major examples include ethyl hexanoate, ethyl acetate, ethyl lactate, and ethyl butanoate [6,55]. Light-aroma Baijiu is typically characterized by ethyl acetate as a major aroma compound, with ethyl lactate and other low-molecular-weight esters modifying its delicate style [10,11,56].

Individual compounds showed clearer differences among aroma types. Ethyl acetate concentrations in groups A, B, and C were 419.629, 249.700, and 355.472 mg/L, respectively. The concentration in A was 68.1% higher than that in B and 18.0% higher than that in C. Its VIP value was 1.039, indicating both high abundance and potential for class discrimination. This result is consistent with the major aroma role of ethyl acetate in light-aroma Baijiu [10,11,56]. Ethyl hexanoate concentrations were 367.805, 1652.044, and 2302.198 mg/L in A, B, and C, respectively. The concentrations in B and C were 4.492 and 6.259 times that in A, and the VIP value was 1.025. The marked enrichment of ethyl hexanoate in B agrees with the established aroma framework of strong-aroma Baijiu, which is dominated by ethyl hexanoate and related short-chain fatty acid esters [6,8,55]. The still higher concentration in C indicates substantial ethyl hexanoate accumulation in the sauce-aroma samples in this batch. It also shows that concentration rankings from a single batch cannot replace general patterns derived from multiple brands and batches. Concentration differences may also be affected by product grade, fermentation round, storage time, and semiquantitative response variation.

Ethyl lactate concentrations in groups A, B, and C were 1134.643, 1854.643, and 8693.685 mg/L, respectively. The concentration in C was 7.662 times that in A and 4.687 times that in B. Despite the large differences among groups, its VIP value was only 0.907 and did not exceed 1. This result shows that VIP is not a direct function of the concentration ratio. PLS-DA considers covariance structure in latent-variable space, within-group variation, and the overall explanatory contribution to class membership. Ethyl lactate may change in parallel with several acids and esters. The resulting information overlap may explain why the model did not assign it a high independent discriminative weight. Ethyl acetate, ethyl lactate, and other esters participate directly in the aroma of sauce-aroma Baijiu [56,57]. They may also affect overall perception by modifying the release of other volatile compounds [17,25].

Several short-chain fatty acids and their esters had higher VIP values. Ethyl pentanoate concentrations in groups A, B, and C were 20.438, 1031.223, and 432.273 mg/L, respectively, with a VIP value of 1.129. Its concentration in B was 50.456 times that in A and 2.386 times that in C. Pentanoic acid concentrations were 22.958, 800.199, and 448.200 mg/L, with a VIP value of 1.133. Butanoic acid concentrations were 170.142, 1272.929, and 1271.748 mg/L, with a VIP value of 1.100. Concentrations in B and C were nearly identical and were approximately 7.48 times that in A. Furfural concentrations were 4.000, 50.000, and 37.090 mg/L, with a VIP value of 1.135. The concentrations in B and C were 12.500 and 9.273 times that in A. Together, these compounds indicate stronger organic acid metabolism, esterification, and accumulation of thermal-reaction products in the strong-aroma and sauce-aroma samples. Key components of sauce-aroma Baijiu generally include esters, acids, alcohols, and aldehydes [7,9,57,58]. Pyrazines are also important contributors to roasted, nutty, and sauce-like aromas [19,20,21,59,60,61].

Higher alcohols showed a different pattern from esters. The concentrations of 3-methyl-1-butanol in groups A, B, and C were 503.296, 287.600, and 128.232 mg/L, respectively. The concentration in A was 3.925 times that in C. This compound was retained as a potential differential variable under the VIP-greater-than-1 screening criterion. Its relative enrichment in light-aroma Baijiu may contribute to alcoholic and fermented aromas. It may also combine with ethyl acetate and other esters to form a more direct aroma profile [10,11,17,18,56]. In contrast, 2-methyl-1-propanol reached 213.545 mg/L in B, compared with 104.786 mg/L in A and 62.068 mg/L in C. Although this compound showed a clear concentration difference, it did not exceed the VIP threshold of 1. The contrasting behavior of these two higher alcohols demonstrates that VIP is not determined by concentration ratios alone. It also reflects covariance with class membership, within-group consistency, and information shared with correlated variables.

The PLS-DA model generated valid VIP scores for 731 of the 732 input compounds. N-nitrosodimethylamine was the only variable without a valid VIP score because it was detected only in group A and provided too few nonzero observations for stable calculation. It was therefore excluded from the final VIP analysis. This exclusion did not alter the ranking of the remaining 731 variables. A VIP value greater than 1 was used as an exploratory criterion for potential differentiation. A total of 366 compounds met this criterion, representing 50.1% of the modeled variables. The selected compounds covered all major chemical classes. Sulfur-containing compounds, aldehydes, and aromatic compounds showed particularly strong representation. This result indicates that compounds occurring at low concentrations or belonging to smaller chemical classes may still provide useful classification information. The VIP distribution was relatively narrow. The separation among the three Baijiu groups was therefore supported by coordinated variation across multiple compound classes rather than by a few dominant markers. VIP was interpreted as a multivariate prioritization index. It was not considered a direct measure of concentration, odor activity, or sensory contribution. All compound-specific VIP values and their potential differential classifications are provided in Supplementary S2.

**Figure 1:**
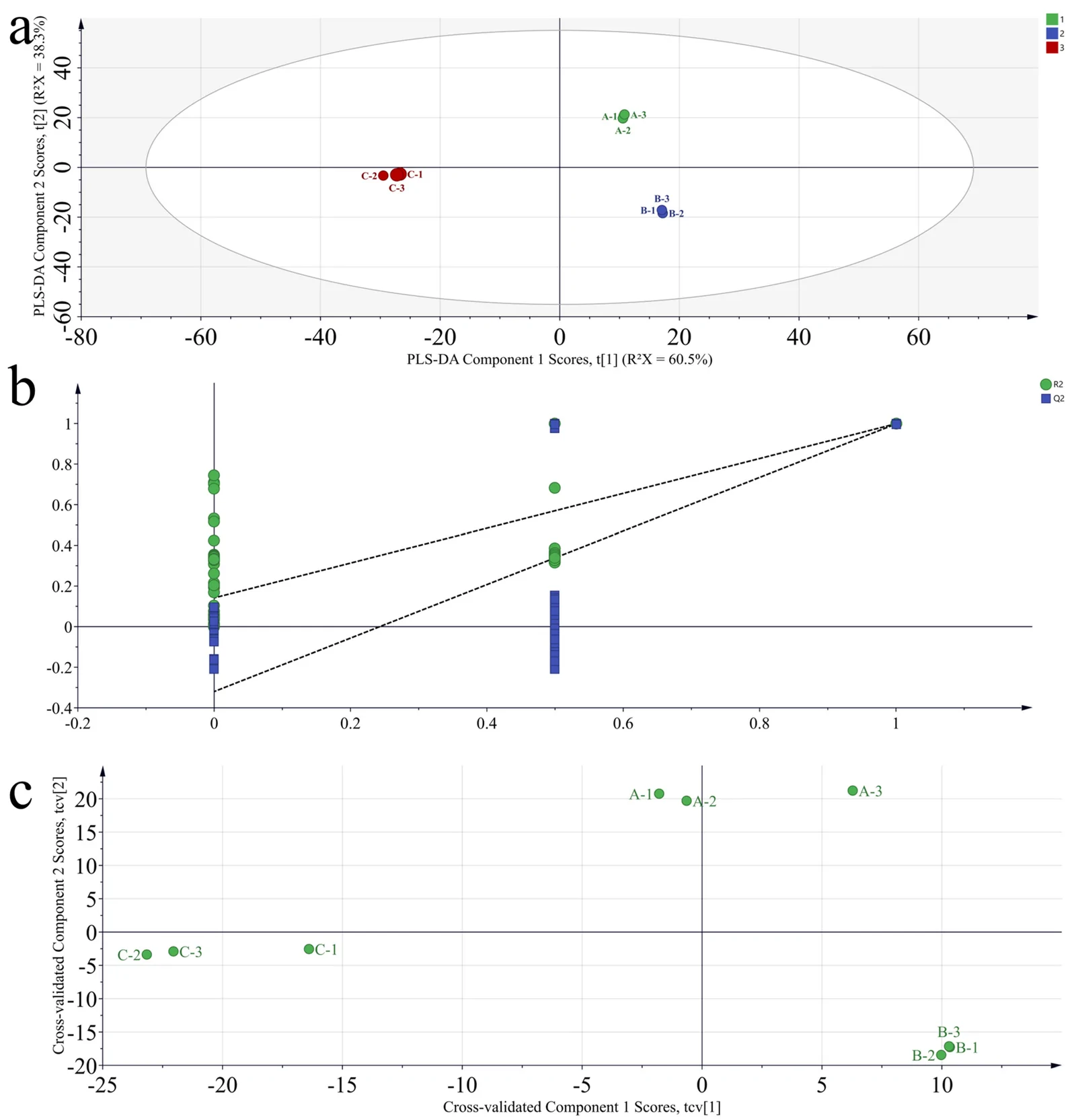
Exploratory PLS-DA analysis based on trace-component concentrations in the 9 analytical observations derived from 3 investigated Baijiu products. Panel (**a**) shows the PLS-DA score plot, in which components 1 and 2 explain 60.5% and 38.3% of the X-matrix variation, respectively. Panel (**b**) shows the 200-permutation test, with R^2^ and Q^2^ intercepts of 0.142 and −0.320. The dashed lines represent the regression lines for R^2^Y (cum) and Q^2^ (cum), respectively. Panel (**c**) shows the internal cross-validated PLS-DA score plot. The observed separation is specific to the current sample set and does not represent population-level validation of discrimination among the 3 Baijiu aroma styles.

Figure 1 shows that PLS-DA components 1 and 2 explained 60.5% and 38.3% of the X-matrix variation, respectively, with a cumulative explained variance of 98.8%. Group A was located in the upper part of the score plot, group B in the lower right, and group C on the left. No overlap was observed among the three groups in the fitted score space. The three replicate analytical preparations within each group clustered closely, indicating repeatability within the investigated product. The model had an R^2^Y of 0.999 and a Q^2^ of 0.998, indicating a strong apparent fit and internal cross-validated separation among the nine observations in the current dataset. The 200-permutation test yielded an R^2^ intercept of 0.142 and a Q^2^ intercept of −0.320. Q^2^ values after random permutation were generally much lower than that of the original model, and no clear random overfitting pattern was observed within this dataset. CV-ANOVA yielded F (8,8) = 5.176 and *p* = 0.0159. The cross-validated score plot retained the spatial separation of groups A, B, and C. All nine observations were classified correctly, producing an internal classification accuracy of 100% and a Fisher probability of 0.0036. These statistics describe only the internal behavior of the fitted model. The nine observations were derived from three commercial products, with one product representing each aroma type and three analytical preparations obtained for each product. They were not independent products or production batches sampled from the broader populations of the three aroma styles. The PLS-DA analysis should therefore be regarded as exploratory. It demonstrates compositional separation among the investigated products and supports variable prioritization within the current dataset. It does not establish external predictive accuracy or robust population-level discrimination among the three Baijiu aroma styles.

Overall, the three Baijiu groups differed markedly in component number, cumulative concentration, and chemical-class composition. Light-aroma Baijiu contained fewer compounds and showed relative enrichment of ethyl acetate and several higher alcohols. Strong-aroma Baijiu contained higher levels of short-chain fatty acid esters and their precursor acids. Ethyl pentanoate, butanoic acid, and pentanoic acid showed strong discriminative effects. Sauce-aroma Baijiu had the broadest compound coverage and highest total abundance. Ethyl lactate, ethyl hexanoate, organic acids, acetals, and lactones formed a broader compositional background. The PLS-DA model provided an exploratory description of compositional separation among the three investigated products. The resulting VIP feature set was used to prioritize variables for subsequent sample-specific analysis. These patterns should not be generalized as population-level differences among the three Baijiu aroma styles without validation using independent products and production batches.

### 3.2. Construction, Parameter Optimization, and Performance Evaluation of the Comprehensive Contribution Ranking Model for Baijiu Aroma Compounds

The literature search covered the Web of Science Core Collection, Scopus, PubMed, and CNKI from January 2002 to December 2025. English search terms included Baijiu, Chinese liquor, Chinese distilled spirit, Chinese spirit, flavor compound, aroma compound, odor-active compound, volatile compound, non-volatile compound, trace component, and metabolite. Method-related terms included GC-MS, GC-O, GC×GC–TOFMS, HPLC, and metabolomics. Chinese search terms covered Baijiu and Chinese distilled spirits, together with terms for flavor components, aroma components, odor-active compounds, volatile components, non-volatile components, trace components, and metabolites. Corresponding analytical terms were also used. Terms within each concept group were connected with OR, and different concept groups were connected with AND. References cited by eligible studies were searched to identify additional sources. Eligible publications were peer-reviewed journal articles in Chinese or English, including original research and reviews. The included studies addressed the identification and quantification of Baijiu aroma and flavor compounds, odor activity, sensory analysis, fermentation metabolism, and analytical techniques. The final dataset comprised 435 literature sources listed in Supplementary S3.

The comprehensive contribution ranking model was developed from a knowledge base of 5572 Baijiu aroma-related compounds. After identifier harmonization, field-completeness checks, and screening for feature computability, 5568 compounds were included in the calculation. RDKit was used to calculate molecular descriptors, including molecular weight, LogP, TPSA, numbers of hydrogen-bond donors and acceptors, rotatable bonds, and ring structures. The dataset is provided in Supplementary S4. Pandas was used for data cleaning, feature integration, and dataset splitting. Model development and result output were completed in Python. The detailed calculations for the comprehensive contribution ranking model are provided in Supplementary S5. Supplementary S5 describes the standardization of five modules covering odor, physicochemical properties, molecular structure, matrix-related properties, and evidence quality. It also describes concentration activation, matrix correction, construction of 23 sample-level features, parameter determination, and performance evaluation. The model contains a compound-level comprehensive contribution ranking component and a sample-level integrated intensity prediction component. Compound scores integrate five categories of information, including odor, physicochemical properties, molecular structure, the Baijiu matrix, and evidence quality. The comprehensive score, contribution proportion, and within-sample rank are calculated for each compound. The score should be interpreted as a dimensionless within-sample prioritization index. A higher score means that a compound receives stronger combined support from the odor, physicochemical, molecular structure, matrix, and evidence modules after concentration activation and matrix adjustment. A lower score indicates weaker integrated support under the same modeled sample conditions. The numerical difference between two compounds describes their separation on this constructed index. It is not a linear sensory scale. For example, a score difference of 0.10 does not mean that one compound produces 10% greater aroma intensity or makes a 10% greater sensory contribution than another. The associated contribution proportion, reported as Contribution_Percent, is obtained by normalizing the Model_Score values so that their sum equals 100% within each modeled sample group. It represents the share of the modeled priority pool assigned to a compound. It does not represent the experimentally measured percentage of perceived aroma. Direct comparisons should therefore be restricted to compounds within the same modeled sample group. Absolute comparisons among Baijiu aroma styles are avoided because their detected compound sets, concentration profiles, and matrix adjustments differ. Closely spaced scores indicate comparable model priorities. Small differences in rank should not be overinterpreted, particularly when the reported P05 to P95 intervals overlap. This interpretation follows established principles for composite indicators. The meaning and stability of such indices depend on normalization, weighting, aggregation, and uncertainty [62,63,64].

**Figure 2:**
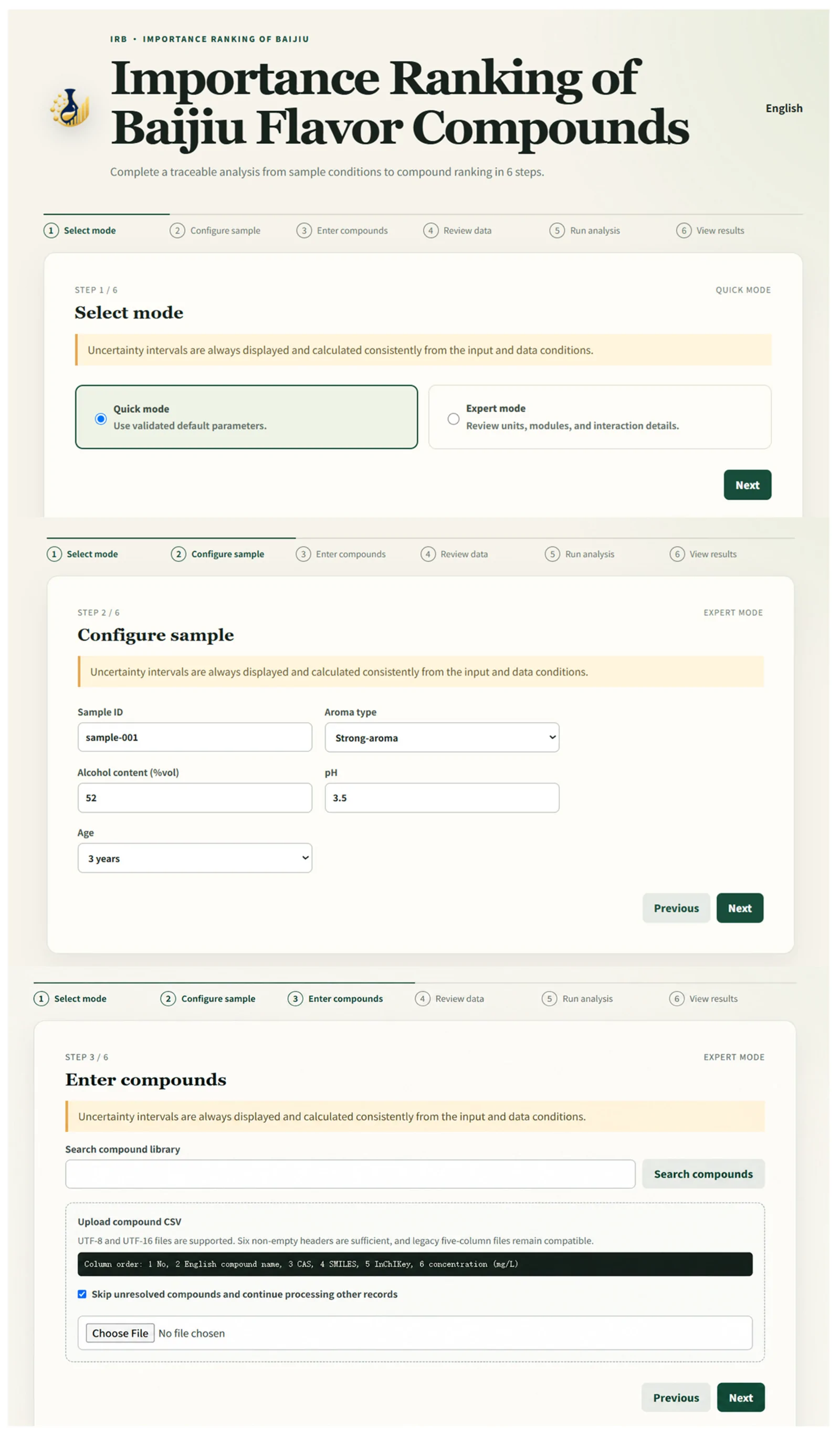
Front-end interface of the importance ranking model for Baijiu aroma compounds.

The sample-level integrated intensity model uses 23 sample-level features as inputs. Feature combinations and model parameters are selected using the validation set, and performance is assessed using an independent test set. The deployed front-end interface is shown in Figure 2. The comprehensive contribution score is used only for the relative ranking of compounds within a specific Baijiu sample. It does not represent the absolute value of their true sensory contributions.

#### 3.2.1. Data Composition and Grouping

Core trace components reported for 12 Baijiu aroma types were used as candidate sources. Aroma-type information and combination rules were applied to construct 240 basic formulation groups. Each group contained 12 compounds and their corresponding reference concentrations. The number 240 refers to 240 independent mixtures rather than 240 individual compounds. Each basic formulation group was defined as a parent formulation for subsequent grouping and data augmentation. Log-normal perturbations centered on the reference concentrations of the 12 compounds in each parent formulation were applied to generate 100,000 sample records. Data were split by parent-formulation family. Records from the same parent formulation were assigned exclusively to the training, validation, or test set. The training set contained 74,980 records from 180 parent-formulation families. The validation set contained 10,008 records from 24 families. The test file contained 15,012 records from 36 families. The reported metrics excluded 6012 development-probe records from the test file, leaving 9000 samples in the formal independent test set. Details are provided in Supplementary S6–S9.

The response variable used in this computational evaluation was a synthetic sensory proxy rather than an experimentally measured sensory score. Each sample was represented by 23 observable variables. These variables comprised five concentration-activated module means, module dispersion, background odor information, two aging terms, two module interaction terms, three summaries of log-transformed concentration, and nine concentration-weighted molecular descriptors. A fixed chemical signal η was calculated from these 23 variables using coefficients specified before model fitting. The complete coefficients and variable order are reported in Supplementary S5. The proxy target was generated as follows.

y = softplus (η + 0.035u_f_ + 0.025u_s_)

In this expression, u_f_ was a standard normal random effect shared by samples derived from the same parent formulation. The term u_s_ was an independently generated standard normal effect specific to each sample. The two random effects were mutually independent. Neither term was supplied to the prediction model. Their inclusion introduced unexplained variation at the formulation and sample levels. It prevented the response from being a fully deterministic copy of the observable chemical signal.

The relationship between η and the 23 predictors was intentional. The synthetic target is therefore not an independent sensory outcome. This benchmark tests whether the computational workflow can recover a predefined chemical mapping in the presence of hidden variation. It does not demonstrate that the mapping represents the sensory response of authentic Baijiu. The resulting R^2^, RMSE, and MAE values are interpreted only as measures of recovery of this synthetic proxy. Independent evidence concerning actual aroma performance was obtained from the subsequent sensory recombination experiment.

Several controls were applied to prevent information leakage during model fitting and evaluation [65,66,67]. The target, u_f_, u_s_, parent-formulation identifier, split label, generator version, random seed, generation time, and generation index were excluded from the predictor matrix. Dataset partitioning was completed at the parent-formulation level before model fitting. The 180 training families, 24 validation families, and 36 test families were mutually exclusive. A direct identifier audit also confirmed that no sample identifier or no missing parent-formulation identifier appeared in more than one partition. Feature scaling parameters were estimated from the training set alone. Candidate feature counts and regularization strengths were selected using validation RMSE. The released fitting procedure did not use test records for feature selection, scaling, coefficient estimation, or parameter selection. The formal test subset was accessed only after the model specification had been fixed. These controls prevent information from the validation or test partitions from influencing model training. They do not remove the designed dependence between the observable features and the synthetic proxy. This distinction defines the scope of the predictive validation and avoids treating a computational recovery test as evidence of sensory accuracy.

#### 3.2.2. Comprehensive Contribution Features and Within-Sample Rankings of Aroma Compounds in Different Baijiu Aroma Types

Aroma-related compounds in the light-aroma, strong-aroma, and sauce-aroma sample groups were scored comprehensively and ranked within each sample group. The light-aroma group yielded 191 valid results. The strong-aroma group yielded 471 valid results, whereas three compounds were excluded because PubChem verification could not be completed. The sauce-aroma group yielded 503 valid results, with seven additional compounds failing verification. Detailed scores are provided in Supplementary S10. Compounds in each group were ranked from highest to lowest comprehensive score, and within-group contribution proportions summed to 100%.

The comprehensive score retained a strong monotonic relationship with concentration but did not reproduce the concentration order completely. Spearman correlation coefficients between score rank and concentration rank were 0.908 for the light-aroma sample, 0.967 for the strong-aroma sample, and 0.960 for the sauce-aroma sample. All correlations were significant at *p* < 0.001. Detailed comparisons of the comprehensive score, concentration ranking, and prior-based OAV reference, including the complete rank positions, Spearman correlation coefficients, and top 20 overlap results, are provided in Table 2. Concentration therefore remained an influential component of the ranking. This result was expected because concentration activation is included in the scoring pathway. The high correlations also indicate that the multi-source framework does not disregard the basic abundance structure of each sample.

Differences became clearer among the highest-priority candidates. The score and concentration rankings shared 11 of the top 20 compounds in light-aroma Baijiu, 10 in strong-aroma Baijiu, and nine in sauce-aroma Baijiu. Thus, nine, 10, and 11 candidates, respectively, were replaced after the additional feature modules were considered. Tetra methylpyrazine and several short-chain acids entered the top 20 score list for the light-aroma sample despite their lower concentration positions. Ethyl butanoate was ranked 11 by the comprehensive score but 110 by concentration in the strong-aroma sample. In the sauce-aroma sample, ethyl octanoate, ethyl dodecanoate, ethyl levulinate, and several other esters received substantially higher priorities than those indicated by concentration alone. These changes arose from the combined effects of odor evidence, molecular characteristics, matrix adjustment, and evidence quality. They show that the score is not a simple rescaling of concentration.

The prior-based OAV reference produced the same ordering as concentration because the common threshold prior of 1.0 mg/L was applied to compounds lacking numerical thresholds. Its correlation coefficients and top 20 overlaps were therefore the same as those obtained for concentration. This result should not be interpreted as agreement with or superiority over a conventional OAV ranking based on independently determined compound-specific thresholds. The identical ordering arose from the imposed common threshold prior. For compounds assigned the same threshold of 1.0 mg/L, OAV was directly proportional to concentration. The prior-based OAV reference therefore functioned as a sensitivity analysis of the missing threshold rule rather than as an independent OAV benchmark. Published work has demonstrated that olfactory thresholds in Baijiu depend on ethanol concentration, the selected reference matrix, and interactions among mixture components [17,18,50,51]. Threshold values obtained from water or a different hydroalcoholic system cannot be transferred without qualification to all three Baijiu matrices.

The comparison supports a restrained justification for the comprehensive score. Concentration remains its quantitative anchor. The additional modules modify the order of candidates that have similar abundance but different odor evidence, structural properties, matrix behavior, or evidence strength. The present results demonstrate a broader prioritization process. They do not establish that the score is superior to concentration or OAV for predicting sensory contribution. Such a conclusion would require parallel recombination models constructed independently from the top 20 compounds selected by each ranking method, followed by blinded sensory comparison. The current recombination experiment evaluates the practical utility of the integrated ranking alone. It therefore provides experimental support for candidate selection but not a direct comparative validation against the two simpler baselines.

**Figure 3:**
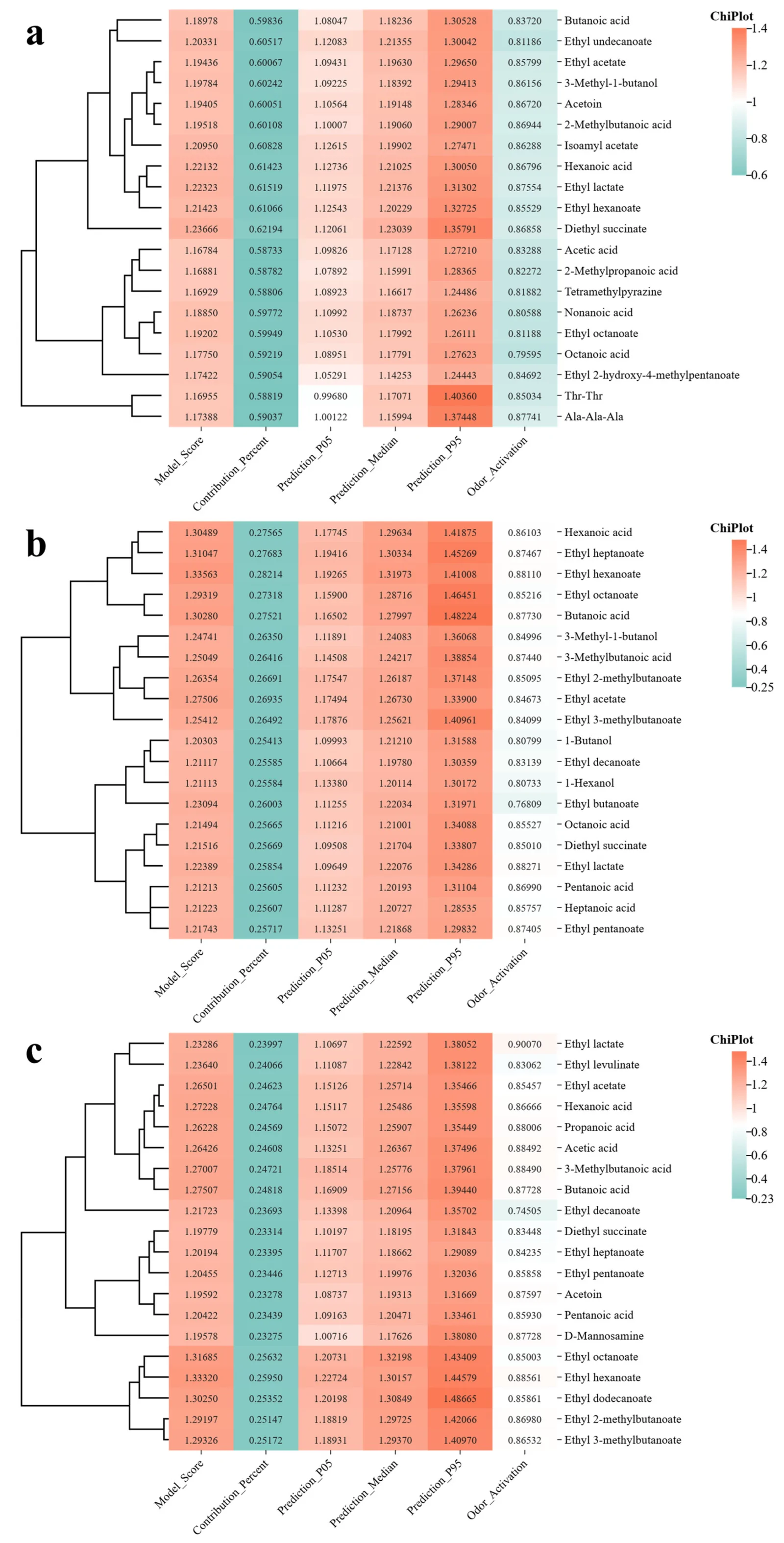
Comprehensive contribution scores and within-group rankings of the top 20 aroma compounds in three Baijiu aroma types. Panel (**a**) shows the scores for light-aroma Baijiu. Panel (**b**) shows the scores for strong-aroma Baijiu. Panel (**c**) shows the scores for sauce-aroma Baijiu.

As shown in Figure 3a, scores for the light-aroma sample ranged from 0.69357 to 1.23666, with a mean of 1.04104 and a median of 1.07419. Scores for the top 20 compounds ranged from 1.16784 to 1.23666, with a cumulative contribution of 12.00023%. Diethyl succinate ranked first with a score of 1.23666 and a contribution of 0.62194%. Ethyl lactate, hexanoic acid, ethyl hexanoate, and isoamyl acetate followed, with scores of 1.22323, 1.22132, 1.21423, and 1.20950, respectively. The top 20 also included typical ester–acid system members such as ethyl acetate, butanoic acid, octanoic acid, ethyl butanoate, and ethyl pentanoate. These compounds formed a highly ranked ester–acid candidate set that was broadly consistent with the delicate, soft, and slightly fruity profile of light-aroma Baijiu. Furthermore, 3-methyl-1-butanol is a higher alcohol that may enhance body and regulate ester volatility. Acetoin is associated with milky and sweet aromas and may improve roundness. Tetramethylpyrazine occurs at a low concentration but has nutty and roasted notes that may contribute to aroma complexity [19,30,56,68,69]. Notably, atypical aroma-related compounds such as Ala-Ala-Ala and Thr-Thr entered the top 20. This result indicates that the ranking was affected by molecular structure, evidence information, and matrix-related features. It represents only relative model priority and does not directly demonstrate actual taste or matrix effects. Studies of complex fermented alcoholic beverages such as beer have combined peptidomics, computational biology, molecular simulation, and modern sensomics to identify umami and bitter peptides. Multidimensional sensory tests were then used to evaluate their effects on taste and overall balance [70,71,72]. However, these findings cannot be extrapolated directly to Baijiu. The functions of Ala-Ala-Ala and Thr-Thr in the present samples require independent experimental validation. Esters and acids were relatively balanced among the top 20 light-aroma compounds. This distribution was consistent with a typical profile dominated by estery aroma with coordinated acidity [10,11,30,56,73]. Alcohols and heterocyclic compounds may further harmonize and refine the flavor [68,69].

Scores for the strong-aroma sample ranged from 0.70644 to 1.33563, with a mean of 1.00508 and a median of 1.01649. Scores for the top 20 compounds ranged from 1.20303 to 1.33563, with a cumulative contribution of 5.27886% (Figure 3b). Ethyl hexanoate ranked first with a score of 1.33563 and a contribution of 0.28214%. Ethyl heptanoate, hexanoic acid, butanoic acid, and ethyl octanoate ranked second to fifth, with scores of 1.31047, 1.30489, 1.30280, and 1.29319, respectively. Medium- and short-chain fatty acid ethyl esters, including ethyl butanoate, ethyl pentanoate, and ethyl nonanoate, ranked highly among the top 20. These compounds contribute the intense fruity and sweet aromas characteristic of strong-aroma Baijiu [6,8,15,29,55]. Fatty acids such as butanoic acid, hexanoic acid, and octanoic acid also ranked highly, forming distinct ester–acid pairs. However, the rankings alone do not demonstrate a dynamic fermentation balance or actual synergistic mechanisms. The top 20 included 11 esters, six acids, and three alcohols. Alcohols such as isoamyl alcohol and 1-propanol did not rank at the top but can regulate aroma release and enhance the body [6,15,29,55]. The matrix correction coefficient for ethyl hexanoate was 1.19239, clearly above 1. Under the specified model parameters, this value produced a relatively high matrix adjustment and indicates that the matrix module affected its ranking. It does not constitute experimental confirmation of a matrix-enhancement effect. Ethyl octanoate and ethyl nonanoate also received high scores, indicating high relative priorities under the specified model. Their actual sensory behavior in a complex matrix still requires experimental validation. Overall, the top 20 strong-aroma compounds formed a structure centered on esters, supported by acids, and modulated by alcohols. This pattern was broadly consistent with the intense, sweet, and balanced aroma characteristics of strong-aroma Baijiu.

Scores for the sauce-aroma sample ranged from 0.69834 to 1.33320, with a mean of 1.02139 and a median of 1.03746. Scores for the top 20 compounds ranged from 1.19578 to 1.33320, and their cumulative contribution was 4.87259%. Ethyl hexanoate ranked first with a score of 1.33320 and a contribution of 0.25950%. Ethyl octanoate, ethyl dodecanoate, ethyl 3-methylbutanoate, and ethyl 2-methylbutanoate completed the top five, with scores of 1.31685, 1.30250, 1.29326, and 1.29197, respectively (Figure 3c). In addition to these esters, the top 20 contained fatty acids such as hexanoic acid, butanoic acid, and decanoic acid. Atypical flavor-related compounds such as acetoin and D-mannosamine were also included [7,9,57,58,60,61,74]. The top 20 contained 12 esters and six acids, which together accounted for 90.00%. They formed a dispersed set of highly ranked candidates. Their actual combined effects require independent experimental validation [16,19,20,21,50,75,76,77]. Acetoin is associated with milky and fermented aromas, and its high ranking indicates that it deserves further attention. However, the proposed bridging effect was not directly verified in this study. D-mannosamine is an amino sugar that also ranked highly, but its actual function in the current aroma system requires independent validation. Several long-chain esters, including ethyl dodecanoate, also received high scores. This result indicates high relative ranking under the specified model rather than confirmed sensory stability. The cumulative contribution of the top 20 was lower for sauce-aroma Baijiu than for the other two aroma types. This result was related to a larger candidate set and a more dispersed contribution distribution. The dispersed score distribution should not be used to infer directly which compound network dominates the authentic Baijiu matrix.

In light-aroma Baijiu, the upper ranks were shared mainly by esters and acids. Representative compounds included diethyl succinate, ethyl lactate, hexanoic acid, ethyl hexanoate, and isoamyl acetate. Their collective prominence suggests a combined ester and acid core rather than dependence on a single marker. Higher alcohols, acetoin, and tetramethylpyrazine extended this pattern across several chemical classes. Some structurally unusual entries, including small peptides, also received relatively high priorities because their molecular and physicochemical features were favorable in the multidimensional model. Direct sensory evidence for these entries remains limited. They should therefore be treated as candidates for targeted verification rather than as confirmed aroma contributors.

The strong-aroma group displayed a more coherent ester and acid architecture. Ethyl hexanoate and related short-chain and medium-chain ethyl esters occurred alongside hexanoic acid and butanoic acid in the high-priority set. This arrangement indicates that the model captured a chemically connected group of aroma precursors, products, and structurally related compounds. Matrix adjustment also changed the relative order of some closely ranked candidates. The ranking thus identifies compounds that merit joint experimental attention. It does not demonstrate that their sensory effects are independent or additive.

The sauce-aroma group showed a broader distribution of modeled priorities. Several ethyl esters and fatty acids remained prominent. Longer-chain esters, acetoin, and D-mannosamine also appeared among the highly ranked candidates. This greater chemical spread suggests that the modeled importance of sauce-aroma Baijiu is distributed across a wider molecular network. The result should not be interpreted as evidence that its aroma can be reproduced using a small set of dominant compounds. It instead identifies a diverse candidate pool whose effects may depend on low-abundance components, matrix conditions, and interactions among compounds.

The top 20 lists for the three groups contained 35 distinct compounds. Light-aroma and strong-aroma Baijiu shared nine compounds, with a Jaccard similarity of 29.03226%. Light-aroma and sauce-aroma Baijiu also shared nine compounds and had the same similarity of 29.03226%. Strong-aroma and sauce-aroma Baijiu shared 14 compounds, with a similarity of 53.84615%. Thus, the latter two groups showed greater consistency in their highly ranked esters and fatty acids. Seven compounds appeared in the top 20 of all three groups. These were diethyl succinate, butanoic acid, hexanoic acid, ethyl lactate, ethyl hexanoate, ethyl acetate, and ethyl octanoate. They formed a shared set of highly ranked candidates. Most were medium- or short-chain esters and their corresponding acids. Previous studies have shown that several have low olfactory thresholds [5,7,8,9,10,11,15,17,68,69,78] and are commonly formed during Baijiu fermentation [5,6,7,8,9]. They may therefore serve as shared candidates across aroma types.

Previous studies provided external references for the model rankings. Gao et al. and Niu et al. used GC-O, quantitative analysis, OAV, and aroma recombination to show that the light-aroma Baijiu profile is jointly formed by multiple esters, acids, alcohols, and low-threshold compounds [10,11]. The top 20 light-aroma compounds in the present model included repeatedly reported aroma-active compounds such as ethyl acetate, ethyl lactate, ethyl octanoate, hexanoic acid, and 3-methyl-1-butanol. The candidate set also overlapped with those reported in recombination studies of aged light-aroma and Xiaoqu light-aroma Baijiu [30,56,73]. The strong-aroma top 20 were centered on ethyl hexanoate, medium- and short-chain fatty acids, and their ethyl esters. This result agreed with molecular sensory studies and recombination-omission experiments [6,15,29,31]. The sauce-aroma top 20 showed less overlap with the literature. Duan et al., Guan et al., and Mou et al. reported that perception of sauce-aroma Baijiu depends not only on high-OAV compounds. It is also affected by trace-component proportions, floral and fruity aroma release, the nonvolatile matrix, and component interactions [9,75,76]. Literature overlap can therefore provide supporting evidence for external consistency but cannot replace sensory recombination for a specific sample.

Overall, the top 20 candidates included several esters, fatty acids, and higher alcohols that have been repeatedly reported in the literature. They also contained a small number of atypical aroma-related compounds. The results represent relative priorities under a specified model and sample condition. They cannot be equated directly with the true sensory contributions of individual compounds or used to determine specific flavor-formation mechanisms. The top 20 compounds from the light-aroma, strong-aroma, and sauce-aroma groups were therefore selected as standards for the subsequent sensory recombination experiment. This procedure was used to evaluate the explanatory ability of each candidate set [29,30,31,32,50,73,75,76,77]. Within-group contribution proportions can be renormalized and combined accordingly during formulation. Comparison of aroma profile, taste structure, and overall similarity between each recombination sample and its original Baijiu can provide an external reference for the model rankings and a basis for further independent validation.

### 3.3. Sensory Recombination Results and Analysis of Model Applicability

The top 20 compounds in sample groups A, B, and C were selected according to the comprehensive contribution rankings. They were added at their internal-standard-based semiquantitative concentrations in the corresponding original samples. Light-aroma recombination sample AA, strong-aroma recombination sample BB, and sauce-aroma recombination sample CC were then prepared. Mean sensory scores from three independent replicates of each original sample were used as references. Aroma profiles and overall similarities were compared with those of the recombination samples.

The same 10 assessors provided overall similarity scores for AA, BB, and CC. The scores were 8.70 ± 1.16 for AA, 9.20 ± 1.03 for BB, and 5.40 ± 1.26 for CC. Their respective 95% confidence intervals were 7.87 to 9.53, 8.46 to 9.94, and 4.50 to 6.30. The Mauchly test did not indicate a significant departure from sphericity, with W = 0.666 and *p* = 0.196. The repeated measures ANOVA showed a significant effect of the recombination model on overall similarity, with F (2,18) = 24.44, *p* < 0.001, and partial η^2^ = 0.731. Holm-adjusted comparisons showed that BB did not differ significantly from AA. The mean difference was 0.50 points, with a 95% confidence interval from −0.41 to 1.41 and an adjusted *p* value of 0.244. AA exceeded CC by 3.30 points, with a 95% confidence interval from 1.91 to 4.69 and an adjusted *p* value of 0.0014. BB exceeded CC by 3.80 points, with a 95% confidence interval from 2.19 to 5.41 and an adjusted *p* value of 0.0014. The strong-aroma recombination sample had the highest overall similarity. The light-aroma sample also received a high score, whereas the sauce-aroma sample scored markedly lower. Within the scope of the present samples, the top 20 model-ranked candidates adequately restored the principal aromas of light-aroma and strong-aroma Baijiu. Their ability to explain the sauce-aroma profile remained limited.

**Figure 4:**
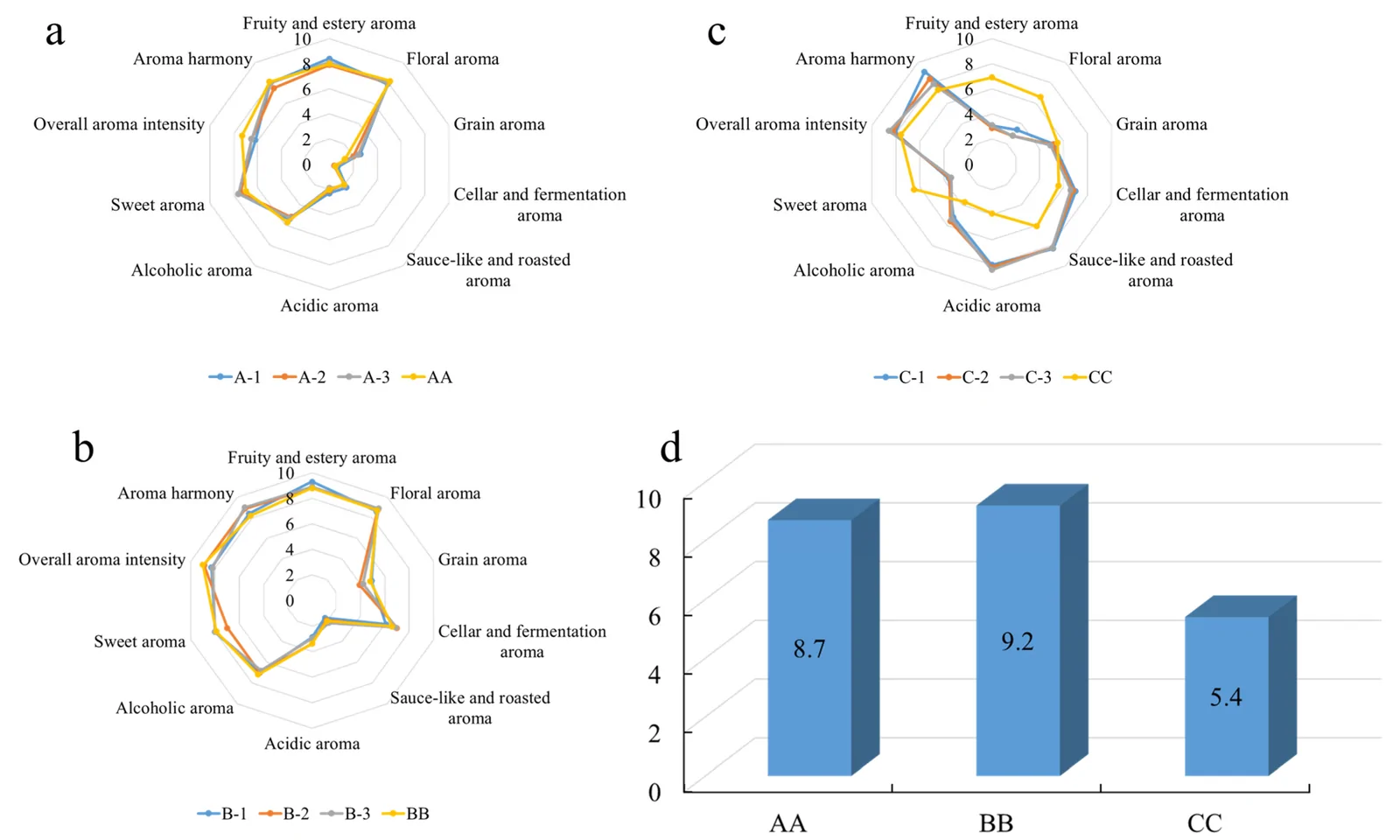
Aroma sensory profiles and reconstruction performance of the original Baijiu samples and their corresponding aroma recombination models. Panels (**a**–**c**) show the aroma sensory profiles of the replicate samples from groups A, B, and C and their corresponding recombination models AA, BB, and CC, respectively. Panel (**d**) shows the overall aroma similarity between each recombination model and its corresponding original Baijiu sample.

The sensory profiles of the three light-aroma original-sample replicates were stable (Figure 4a). The Pearson correlation coefficient between AA and the mean profile of the original sample across 10 sensory dimensions was 0.988. The mean absolute deviation was 0.377 points, and the RMSE was 0.499 points. Fruity and estery aroma scores were 8.13 for the original sample and 8.00 for AA. Floral aroma scores were 7.97 and 8.20, pit and fermentation aroma scores were 0.53 and 0.50, sauce-like and roasted aroma scores were 2.13 and 2.00, and acidic aroma scores were 2.10 and 2.00, respectively. These major attributes showed substantial overlap. The main deviations occurred in grain aroma and overall aroma intensity. The grain aroma of AA was 1.03 points lower than the mean of the original sample, whereas its overall aroma intensity was 0.90 points higher. These deviations did not change the overall structure of the light-aroma sample, which was dominated by fruity, estery, floral, and sweet aromas and had weak pit and sauce-like aromas. The candidate ranking for the light-aroma sample therefore received strong sensory support.

The strong-aroma sample showed the most stable recombination performance among the three aroma types (Figure 4b). The Pearson correlation coefficient between BB and the mean profile of the original sample was 0.992. The mean absolute deviation was only 0.277 points, and the RMSE was 0.330 points. When only eight specific aroma attributes were considered, the mean absolute deviation decreased further to 0.208 points. These attributes were fruity and estery aroma, floral aroma, grain aroma, pit and fermentation aroma, sauce-like and roasted aroma, acidic aroma, alcoholic aroma, and sweet aroma. Scores for the fruity and estery aroma, floral aroma, and pit and fermentation aroma of BB were 8.80, 8.70, and 6.60, respectively. The corresponding mean scores for the original sample were 9.00, 8.77, and 6.67. Sauce-like and roasted aroma differed by only 0.07 points. The largest deviations were observed for aroma harmony, overall aroma intensity, and grain aroma, with differences of 0.57, 0.53, and 0.47 points, respectively. None of these differences substantially changed the primary aroma structure. Together with the overall similarity score of 9.20, these results indicate that the top 20 candidates provided strong recombination support for the fruity, estery, floral, pit, and overall aroma intensity characteristics of strong-aroma Baijiu in the present samples.

The sauce-aroma sample showed a different pattern (Figure 4c). The Pearson correlation coefficient between CC and the mean profile of the original sample across 10 sensory dimensions was only −0.060. The mean absolute deviation was 2.213 points, and the RMSE was 2.576 points. When only eight specific aroma attributes were considered, the mean absolute deviation increased to 2.529 points. Acidic aroma and sauce-like and roasted aroma were prominent in the original sample, with mean scores of 8.20 and 8.23. The corresponding scores for CC were only 3.90 and 6.10, representing decreases of 4.30 and 2.13 points. Fruity and estery aroma increased from 3.03 in the original sample to 6.90 in CC. Floral aroma increased from 3.00 to 6.60, and sweet aroma increased from 3.50 to 6.50. Alcoholic aroma and pit and fermentation aroma decreased by 1.70 and 1.20 points, respectively. Although the overall aroma intensity of CC was only 0.73 points below the mean of the original sample, the proportions among aroma attributes changed markedly. Aroma harmony also decreased from 8.47 to 7.30. Therefore, the recombination sample did not simply lack aroma intensity. Instead, it showed a structural mismatch characterized by excessive fruity, floral, and sweet aromas and insufficient acidic, sauce-like, and roasted aromas (Figure 4d).

Within-assessor comparisons provided further statistical support for these profile differences. The paired difference was defined as the recombination score minus the assessor-specific mean of the three original sample evaluations. After Holm adjustment across the 10 sensory dimensions, no attribute differed significantly between AA and its original light-aroma sample. No significant attribute difference was found between BB and its original strong-aroma sample. The minor deviations observed for these two recombination models should therefore be interpreted as descriptive variation rather than confirmed sensory effects. A different result was obtained for CC. Fruity and estery aroma increased by 3.87 points, with a 95% confidence interval from 2.81 to 4.93 and an adjusted *p* value below 0.001. Floral aroma increased by 3.60 points, with a 95% confidence interval from 2.56 to 4.64 and an adjusted *p* value below 0.001. Sweet aroma increased by 3.00 points, with a 95% confidence interval from 1.56 to 4.44 and an adjusted *p* value of 0.007. Acidic aroma decreased by 4.30 points, with a 95% confidence interval from −5.09 to −3.51 and an adjusted *p* value below 0.001. Sauce-like and roasted aroma decreased by 2.13 points, with a 95% confidence interval from −3.10 to −1.17 and an adjusted *p* value of 0.005. Alcoholic aroma decreased by 1.70 points, with a 95% confidence interval from −2.60 to −0.80 and an adjusted *p* value of 0.011. Differences in grain aroma, pit and fermentation aroma, overall aroma intensity, and aroma harmony did not remain significant after adjustment. The confidence intervals show that the weaker reconstruction of CC arose from changes in aroma proportions rather than a uniform loss of overall intensity.

Previous studies have shown that aroma recombination can validate the screening of key compounds. However, recombination performance depends on compound coverage and interactions [29,30,31,73]. Li et al. identified 32 important odorants in Xiaoqu light-aroma Baijiu. Their recombination sample had an aroma profile similar to that of the original Baijiu, showing that the principal aroma of light-aroma Baijiu can be reproduced using a relatively limited number of key components [73]. Chen et al. identified 152 aroma-active compounds in strong-aroma Baijiu. Their recombination experiment reproduced most sensory attributes and demonstrated important contributions from ethyl acetate, ethyl hexanoate, hexanoic acid, ethyl pentanoate, and other compounds [29]. These findings agree with the high-profile correlations and overall similarities obtained for AA and BB in the present study.

The sensory formation mechanism of sauce-aroma Baijiu is more complex [50,75,77]. Guan et al. reported that more than 500 aroma compounds can be detected in sauce-aroma Baijiu. The sensory contribution of an individual compound is not linearly related to its concentration or OAV [75]. The proportions and coexistence of trace components also affect overall aroma balance [50,75,77]. Mou et al. found that the floral and fruity aroma of sauce-aroma Baijiu is not determined solely by esters. Acetals can improve aroma harmony, and aldehydes can affect the release of floral and fruity compounds [76]. Zhou et al. further showed that adding nonvolatile components made the fruity, sweet, and acidic aromas of strong-aroma recombination samples more similar to those of the original Baijiu. This finding indicates that the Baijiu matrix modifies the release and perceived intensity of volatile compounds [31].

The present results suggest that the top 20 sauce-aroma candidates restored overall aroma intensity to some extent but did not preserve the proportions among acidic aroma, sauce-like and roasted aroma, and floral and fruity aroma. Some lower-ranked compounds with synergistic, inhibitory, or matrix-modulating effects may have been excluded from the recombination system. Differences between the hydroalcoholic model matrix and the authentic sauce-aroma Baijiu matrix may also have enhanced fruity and estery aromas while weakening acidic and sauce-like aromas. The relative rankings for the light-aroma and strong-aroma samples received strong sensory support, with the strong-aroma sample showing the greatest stability. The current sauce-aroma model provides candidate leads, but the sensory interpretability of its rankings requires improvement. Future parameter optimization should strengthen aroma-type-specific prior information for sauce-aroma Baijiu [16,75,76]. Greater weight should also be assigned to compound interactions, low-OAV components, and matrix effects [50,77]. This strategy would reduce the risk of using highly ranked individual compounds to represent the overall aroma of a complex Baijiu matrix.

The triplicate analytical measurements provided an internal assessment of repeatability for representative compounds included in one or more recombination models. The corresponding means and standard deviations are reported in Supplementary S1. Across ethyl acetate, ethyl octanoate, 3-methyl-1-butanol, butanoic acid, hexanoic acid, acetoin, ethyl hexanoate, diethyl succinate, acetic acid, and ethyl lactate, the relative standard deviations ranged from 0.73% to 15.15%. The RSD values for ethyl acetate were 14.53%, 3.92%, and 2.80% in groups A, B, and C, respectively. The corresponding values for ethyl hexanoate were 9.63%, 8.73%, and 13.67%. Those for ethyl lactate were 2.34%, 3.23%, and 15.15%. These results indicate that the peak-area ratios were reasonably repeatable under the present analytical conditions. They do not establish the quantitative trueness of the reported concentrations. An analyte-specific calibration curve was not available for every detected compound. A relative response factor of 1 was therefore assumed during semiquantification. The selection of structurally similar internal standards can reduce major response mismatches. It cannot fully correct compound-dependent differences in extraction recovery, inlet transfer, ionization efficiency, fragmentation behavior, and detector response. Such differences are recognized sources of uncertainty in nontarget and semiquantitative GC-based analysis [79,80]. Any systematic overestimation or underestimation was carried into the recombination formulation because the added amounts were derived directly from the semiquantitative concentrations. The sensory consequence may not be proportional to the concentration error. Aroma responses depend on odor thresholds, matrix release, perceptual saturation, and interactions among compounds. A modest dosing error in a highly potent odorant may therefore alter the balance of an aroma profile. The close agreement obtained for models AA and BB suggests that the estimated concentrations were sufficient to reproduce their dominant sensory patterns at the profile level. It should not be regarded as compound-specific quantitative validation. The weaker agreement for model CC may partly reflect semiquantitative dosing uncertainty. It cannot be attributed solely to compound ranking, omitted odorants, or aroma interactions. The recombination results therefore support the plausibility of the selected candidate sets, but they do not demonstrate exact concentrations or absolute sensory contributions.

## 4. Discussion and Limitations

### 4.1. Biological and Technological Basis for the Lower Reconstruction Accuracy of Sauce-Aroma Baijiu

The aroma recombination results were not equally consistent across the three Baijiu aroma types. The light-aroma and strong-aroma recombination samples closely resembled their corresponding original samples. A different outcome was obtained for sauce-aroma Baijiu. The overall similarity score of CC was 5.40 ± 1.26. Its sensory-profile correlation with the original sample was −0.060. Acidic aroma and sauce-like and roasted aroma were insufficient. Fruity, floral, and sweet aromas were overexpressed. However, the overall aroma intensity of CC was only 0.73 points lower than that of the original sample. The discrepancy was therefore not caused simply by an insufficient amount of volatile material. It arose mainly from an incorrect balance among sensory attributes. This experiment directly demonstrates that the 20 selected compounds, when added at their semiquantitative concentrations to the hydroalcoholic matrix used in this study, were insufficient to reproduce the aroma structure of the investigated sauce-aroma Baijiu.

A biological explanation can be proposed for this result, although it was not tested directly in the present study. Sauce-aroma Baijiu is produced through open solid-state fermentation. Its process includes high-temperature Daqu preparation, high-temperature stacking, repeated pit fermentation, staged distillation, storage, and blending. Each operation creates a distinct ecological and chemical environment. Daqu introduces microorganisms and enzymes. The production environment, tools, pit surfaces, and fermentation vessels provide additional microbial sources. Temperature, acidity, ethanol content, oxygen availability, moisture, and substrate composition subsequently impose strong selection pressures on the microbial community [81,82].

Microbial succession also changes across fermentation stages and production rounds. Bacteria, yeasts, and filamentous fungi participate in the conversion of starch, proteins, lipids, and intermediate metabolites. Their metabolic activities generate acids, alcohols, esters, aldehydes, ketones, pyrazines, sulfur-containing compounds, nitrogen-containing compounds, and numerous aroma precursors. Studies of sauce-aroma Baijiu have shown that microbial composition and volatile profiles vary among fermentation rounds, spatial positions, and production regions [83,84,85]. These changes can produce chemically distinct base Baijiu fractions. Subsequent blending and aging integrate these fractions into the final product. The characteristic aroma may consequently depend on a distributed chemical network rather than a small group of independently acting odorants.

The role of microbial ecology in the present reconstruction difference should be interpreted carefully. Microorganisms do not directly participate in sensory recombination after distillation. Their relevance is indirect. A diverse and temporally changing microbiota can create a broader range of odorants, precursors, and nonvolatile metabolites. It can also establish distinctive proportions among these components. A ranking model restricted to detected compounds and individual compound features may not fully represent this process-generated chemical organization. The present study did not analyze Daqu, fermented grains, production environments, or microbial communities. It therefore cannot establish a causal relationship between specific microorganisms and the lower similarity of CC. Microbial and technological complexity remains a literature-supported explanation that requires direct verification using paired microbiome, metabolome, and sensory data.

### 4.2. Effects of Compound Interactions, Low-OAV Components, and the Baijiu Matrix

The current ranking model mainly evaluates compounds through their individual odor evidence, molecular descriptors, physicochemical properties, concentration activation, matrix adjustment, and evidence quality. It does not derive explicit pairwise or higher-order sensory interaction terms from controlled mixture experiments. This limitation is particularly relevant to sauce-aroma Baijiu. Aroma perception is not a simple sum of the intensities of isolated compounds. Odorants can produce additive, synergistic, masking, suppressive, or qualitatively new perceptual effects [86]. A highly ranked ester may therefore contribute strongly when evaluated alone but become less prominent in the presence of acids, higher alcohols, aldehydes, or other esters. Conversely, a compound with moderate individual importance may alter the perception of an entire aroma group.

A low OAV should not be treated as proof of sensory inactivity. OAV is calculated from the concentration of an individual compound and its odor threshold in a specified medium. It does not describe how that threshold changes in a complex mixture. Several compounds with OAV values below 1 may collectively reinforce a common sensory note. They may also change the release, recognition, or masking of compounds with higher OAV values. Some lower-ranked aldehydes, acetals, pyrazines, sulfur-containing compounds, and nitrogen-containing compounds may be relevant in this context. Their exclusion from the top 20 set could alter roasted, acidic, fermented, floral, or fruity attributes even when their individual concentrations are low. This interpretation remains a hypothesis because the present experiment did not perform targeted addition or omission tests for the excluded low-OAV compounds.

The simplified recombination matrix creates another limitation. The ethanol concentration was matched to that of the original sauce-aroma sample, and the selected acids were added at their measured concentrations. However, the full nonvolatile composition of the authentic Baijiu was not reconstructed. Polyols, peptides, phenolic compounds, residual organic acids, trace salts, and other fermentation-derived metabolites can modify volatile solubility and gas–liquid partitioning. They may retain some compounds in the liquid phase while promoting the release of others. Such changes affect headspace concentration and perceived aroma even when the amount added remains constant [87]. The authentic matrix may also influence odor thresholds and perceptual integration.

The excessive fruity and floral aromas in CC could therefore reflect the absence of suppressive odorants, inadequate retention of esters, or the omission of nonvolatile matrix components. The insufficient acidic and sauce-like aromas could arise from incomplete coverage of trace odorants or from missing interactions among several chemical classes. These explanations are chemically plausible. They were not isolated experimentally in this study. The current data cannot determine which mechanism was dominant. Future work should compare hydroalcoholic models with deodorized authentic Baijiu matrices and reconstructed nonvolatile fractions. Sequential omission and addition experiments are also needed. Factorial mixture designs could quantify interactions between esters, acids, alcohols, aldehydes, acetals, pyrazines, and sulfur-containing compounds. These experiments would allow interaction parameters to be estimated from sensory evidence and incorporated into later versions of the model.

### 4.3. Scope of Validation and Separation of Experimental Evidence from Model-Generated Hypotheses

The evidence obtained in this study should be separated into three levels. The analytical experiment established which compounds were detected and provided internal-standard-based semiquantitative concentrations. The computational workflow generated relative priorities for compounds within each sample group. The sensory experiment tested whether the 20 highest-ranked candidates could reconstruct the aroma profile under the specified formulation conditions. These are experimentally supported outcomes of the present study.

The score itself is not an experimentally measured sensory contribution. A high score indicates that a compound has strong integrated support under the defined feature system. It does not demonstrate that the compound independently causes a specific aroma attribute. The proposed influences of microbial succession, low-OAV compounds, nonvolatile metabolites, and compound interactions are also not direct experimental findings of this study. They are mechanistic hypotheses developed from the reconstruction pattern and the available literature. The present results identify where the model performed well and where additional mechanisms may be required. They do not prove those mechanisms. The PLS-DA findings require a similarly restricted interpretation. The high R^2^Y and Q^2^ values and the 100% internal classification describe separation among nine observations derived from three commercial products. They do not represent validation using independent products or production batches. The analysis therefore supports exploratory visualization and variable prioritization for the current sample set. It does not support population-level discrimination among light-aroma, strong-aroma, and sauce-aroma Baijiu. The OAV comparison has a separate but related limitation. The common threshold prior of 1.0 mg/L made the prior-based OAV order directly dependent on concentration. Consequently, this reference cannot serve as an independent OAV benchmark and cannot support a claim that the comprehensive score is superior to a conventional OAV ranking based on compound-specific thresholds.

The predictive performance obtained using the synthetic target should be interpreted within the same boundary. It demonstrates that the sample-level algorithm can reproduce the defined synthetic proxy under the applied data-generating scheme. It does not establish equivalent accuracy for human aroma perception. Sensory recombination provides a more direct external assessment. The high similarities obtained for AA and BB offer sample-specific sensory support for the rankings generated for the investigated light-aroma and strong-aroma samples. The lower similarity of CC reveals a clear boundary of applicability. The proposed approach has therefore not been comprehensively validated across all Baijiu aroma styles. It should not be presented as universally transferable to different producers, regions, batches, aging periods, or production systems.

Further validation should use independent samples from multiple producers, production batches, geographical regions, and storage periods. Stable-isotope dilution analysis could reduce uncertainty in the concentrations of representative compounds. Recombination should be performed in both simplified and authentic matrices. Controlled omission, addition, and mixture experiments are needed to separate individual effects from interaction effects. Microbial metagenomics, meta transcriptomics, and metabolomics could connect fermentation ecology with the formation of candidate odorants and precursors. Model weights and aroma-type priors should then be calibrated against authentic sensory endpoints. These steps would extend the current workflow from exploratory prioritization toward externally validated sensory prediction without replacing evaluation by trained assessors.

## 5. Conclusions

This study developed an integrated workflow that connects curated chemical knowledge, comprehensive volatile analysis, multi-source molecular features, computational ranking, and sensory recombination. Its main contribution is not the generation of an absolute numerical measure of aroma contribution. Instead, the workflow provides an interpretable means of prioritizing compounds that warrant experimental verification within a specific Baijiu sample. It also reduces a chemically extensive candidate space to a more manageable set for subsequent sensory investigation. The successful reconstruction of the principal aroma structures of the investigated light-aroma and strong-aroma samples supports the practical value of this strategy. The poorer reconstruction of sauce-aroma Baijiu is equally informative. It shows that reproducing overall aroma intensity does not necessarily restore the internal balance among aroma attributes. It also reveals an important boundary of the current model when applied to highly complex fermented beverages.

Instrumental analysis, computational prediction, and human sensory evaluation provide different forms of information. Chromatographic analysis describes which volatile compounds are present and estimates their abundance. Molecular descriptors and physicochemical properties help explain how compounds may behave within a given chemical environment. Computational models organize this information and identify candidates that may otherwise receive limited experimental attention. Trained assessors evaluate the integrated perceptual outcome. This outcome cannot be inferred completely from chemical composition alone. Aroma perception depends on concentration and odor thresholds, but it is also shaped by matrix-dependent release, interactions among odorants, and the collective effects of low-OAV compounds. Synergistic and antagonistic relationships may strengthen, suppress, or reshape a sensory attribute. Nonvolatile components and fermentation-derived metabolites may further modify volatile partitioning and perception. Sensory recombination therefore serves as a necessary bridge between computational priority and perceptual relevance.

The proposed approach should be viewed as a complement to sensory evaluation rather than a replacement for it. The comprehensive score represents relative model priority within a sample. It does not quantify the true sensory contribution of an individual compound. The sample-level prediction model was fitted to a defined synthetic proxy target. Its performance cannot be interpreted as direct evidence of sensory accuracy in authentic Baijiu. The PLS-DA results are likewise exploratory and are limited to separation among the three investigated products and their replicate analytical preparations. Their high internal performance measures do not establish robust discrimination among Baijiu aroma styles at the population level. The prior-based OAV reference was also not independent of concentration because unavailable thresholds were replaced by a common value. The comparison therefore does not demonstrate superiority over a conventional OAV ranking based on independently determined thresholds. Other limitations include the restricted experimental sample set, uncertainty in semiquantitative concentrations, selection of only the highest-ranked candidates, use of a simplified hydroalcoholic recombination matrix, and the absence of experimentally derived interaction terms. These limitations are particularly important for sauce-aroma Baijiu. Its characteristic aroma may depend on a broad network of volatile and nonvolatile components rather than a small group of dominant odorants.

Future work should include independent samples from different producers, production batches, geographical regions, and storage periods. Quantitative analysis using isotopically labeled internal standards would improve the accuracy of aroma recombination. Controlled addition, omission, and mixture experiments are needed to distinguish individual effects from synergistic or suppressive interactions. Authentic or reconstructed nonvolatile matrices should also be incorporated. Model weights and aroma-type priors could then be calibrated using experimentally obtained sensory data. Integration with microbial, metabolic, and fermentation-process information may further connect aroma prediction with the biological origins of flavor. With these improvements, the workflow could support aroma characterization, quality control, product authentication, fermentation optimization, and sensory prediction. Its general framework may also be extended to other fermented beverages in which chemical complexity and human perception must be considered together.

## Figures and Tables

**Table 1:** Definitions of the sensory attributes.

Evaluation Dimension	Definition	Typical Aroma References
Fruity and estery aroma	Intensity of fruit-like aromas formed by esters and related volatile compounds. Clarity, naturalness, and complexity were considered.	Apple, pear, banana, pineapple, peach, citrus, ripe fruit, preserved fruit, and characteristic estery aroma
Floral aroma	Soft, fresh, sweet, or intense flower-like aromas. Floral clarity and integration with other aromas were considered.	Rose, jasmine, osmanthus, acacia flower, violet, and honey-like floral aroma
Grain aroma	Cereal aromas formed by brewing materials and cooking. Naturalness, cleanliness, and maturity were considered.	Sorghum, corn, wheat, rice, rice husk, steamed grain, cooked cereal, and sweet grain aroma
Pit and fermentation aroma	Complex aromas produced by solid-state fermentation, the fermentation-pit environment, and microbial metabolism. Richness, typicality, and cleanliness were considered.	Pit mud, fermented grains, distillers’ grain aroma, starter aroma, fermented fruit aroma, lactic fermentation aroma, and aged notes
Sauce-like and roasted aroma	Sauce-like, toasted, and roasted aromas formed during high-temperature starter production, stacking fermentation, and thermal reactions. The aroma should be distinct without excessive scorching.	Sauce-like aroma, roasted grain, nuts, coffee, cocoa, caramel, toast, and mild smoky aroma
Acidic aroma	Fermented acidic aroma formed by volatile acids and their interactions with other components. Freshness, softness, and sharp irritation were considered.	Acetic-acid-like aroma, lactic fermentation aroma, fruity acidity, aged-vinegar aroma, and mild fatty-acid notes
Alcoholic aroma	Alcoholic and full-bodied aromas and volatile irritation produced by ethanol and higher alcohols. Purity, softness, and cleanliness were considered.	Clean alcoholic aroma, higher-alcohol aroma, fermented alcoholic aroma, and mild solvent-like or pungent notes
Sweet aroma	Intensity of aromas associated with sweetness rather than actual taste. Roundness and the ability to enhance fullness were considered.	Honey, caramel, vanilla, maltose, ripe fruit, sweet cream, and syrup-like aroma
Overall aroma intensity	Overall strength of all aroma stimuli without separating specific aroma classes. Fullness, concentration, and persistence were emphasized.	A score of 0 indicates almost no aroma, and a score of 10 indicates extremely high overall aroma intensity
Aroma harmony	Degree of integration among aroma attributes. Excessive dominance, mutual masking, irritation, and disorder were considered.	A high score indicates natural transitions among aromas, appropriate proportions, and no obvious discordant notes
Overall similarity	Consistency between a recombination sample and its corresponding original Baijiu in dominant aromas, intensity, attribute proportions, and overall profile.	A score of 0 indicates complete difference, and a score of 10 indicates a highly consistent aroma profile

**Table 2:** Agreement between comprehensive score ranking and simpler reference rankings.

Aroma Type	Scored Compounds	Spearman ρ with Concentration Rank	*p* Value	Shared Compounds in the Top 20	Spearman ρ with Prior-Based OAV Rank	Shared Compounds in the Top 20
Light aroma	191	0.908	<0.001	11	0.908	11
Strong aroma	471	0.967	<0.001	10	0.967	10
Sauce aroma	503	0.96	<0.001	9	0.96	9

Table note: Concentration rankings were obtained by sorting mean semiquantitative concentrations in descending order. Compound-specific numerical odor thresholds were unavailable in the released scoring artifact. The model therefore applied a common threshold prior of 1.0 mg/L. The resulting prior-based OAV ranking was identical to the concentration ranking and does not represent an independent conventional OAV analysis.

## Data Availability

The original contributions presented in the study are included in the article/Supplementary Materials, further inquiries can be directed to the corresponding authors.

## Supplementary Materials

The following supporting information can be downloaded at https://www.mdpi.com/article/10.3390/foods15173043/s1: Supplementary S1: Concentration, Structural Information, and Internal Standard Molecular Similarity Parameters of Trace Components in Three Types of Aroma Baijiu; Supplementary S2: VIP Values and Potential Differential Determination of Trace Components in the PLS-DA Model; Supplementary S3: Literature Sources for the Construction of a Knowledge Base of 5572 Flavor-Related Compounds in Baijiu; Supplementary S4: 5568 Baijiu Flavor-Related Compounds and Characteristic Parameters Used in Model Construction; Supplementary S5: Calculation Method and Parameter Description of the Comprehensive Contribution Ranking Model for Baijiu Aroma Compounds; Supplementary S6: Model Training Set; Supplementary S7: Model Independent Test Set; Supplementary S8: Model Validation Set; Supplementary S9: Formal Evaluation Subset of Model Test Set; Supplementary S10: Summary of Model Results for Three Types of Aroma Samples; Supplementary S11: Participant Information Sheet and Informed Consent Form; Supplementary S12: IRB-Importance Ranking of Baijiu.
