# Supplementary material for: Construction of a Comprehensive Contribution Ranking Model for Baijiu Aroma Compounds Based on Multi-Source Molecular Feature Fusion and Validation by Aroma Recombination
Source: Foods. 2026 Aug 28;15(17):3043. doi: 10.3390/foods15173043 (PMC13565323; doi:10.3390/foods15173043)
Supplement: Supplementary file 1 [file foods-15-03043-s001.zip › Supplementary S11 Participant Information Sheet and Informed Consent Form.pdf]

BEIJING TECHNOLOGY AND BUSINESS UNIVERSITY

Participant Information Sheet and Informed Consent Form

Construction of an Integrated Contribution Model for Baijiu Flavor Compounds Based on Multi-Source Molecular Feature Fusion and Sensory Reconstruction Validation

Important notice

This study involves orthonasal aroma evaluation only. All samples are for smelling only and must not be tasted or swallowed. Please read this document in full and ask any questions before deciding whether to participate.

Dear participant,

You are invited to take part in a sensory study of Baijiu aroma. Before deciding, please read this document carefully. It explains the purpose of the study, the evaluation procedure, possible discomforts, privacy protection, and your rights. You may ask the research team any questions and decide freely after you have understood the information. This is a non-clinical, minimal-risk food sensory study. It does not involve treatment and is not intended to assess your health.

1 Background and purpose

This study will integrate molecular structure, physicochemical properties, aroma annotations, model scores, and other molecular features to construct an integrated contribution model for Baijiu flavor compounds. The 20 highest-scoring compounds will be selected separately from Baijiu samples A, B, and C. Each compound will be added at its internal-standard-based semi-quantitative concentration to a food-grade ethanol and ultrapure-water matrix matched to the alcohol content of the corresponding original sample, producing reconstructed samples A-A, B-B, and C-C. Their aroma profiles will be compared with those of the original samples A, B, and C. The sensory results will be used to test whether the model-selected compounds retain the principal aroma structure of each Baijiu. This validation does not imply that 20 compounds can completely reproduce the original Baijiu or that the semi-quantitative concentrations are absolute concentrations.

2 Participants and eligibility

The study plans to recruit 10 adult assessors with experience in alcoholic-beverage sensory evaluation, including 5 men and 5 women, from the sensory laboratory of Beijing Technology and Business University. Participants should understand a 0-to-10 intensity scale, have normal olfactory function, and be able to record their judgments independently.

- Be at least 18 years old, participate voluntarily, and be able to provide written informed consent.
- Have no severe cold, acute rhinitis, nasal obstruction, or other condition that has clearly affected smell during the previous 7 days.
- Have no known alcohol allergy or marked intolerance to the odor of high-strength alcohol.
- Have no respiratory condition that may be triggered by alcohol vapor or strong odors.
- Not be using medication that clearly affects olfactory judgment. As a precaution, people who are pregnant or breastfeeding will not normally be enrolled.

3 Study procedures

The samples comprise the original Baijiu samples A, B, and C and their corresponding reconstructed samples A-A, B-B, and C-C. The alcohol contents of A and A-A are 53% vol, those of B and B-B are 52% vol, and those of C and C-C are 53% vol. The pH of the reconstructed matrices will not be adjusted separately. Selected acids and all other selected compounds will be added at their measured internal-standard-based semi-quantitative concentrations. A 15 mL aliquot of each sample will be placed in a covered standard Baijiu sensory glass and equilibrated at 20 ± 1 °C. Samples will be identified by random three-digit codes and presented in randomized order. Each assessor will complete three independent evaluation rounds with adequate rest between rounds.

Smelling only

The evaluation is limited to orthonasal aroma. You will briefly remove the lid, smell the headspace above the glass without touching the liquid, and replace the lid after making your judgment. Reconstructed samples contain research-grade compound standards and are for smelling only. They must not be tasted, sipped, or swallowed.

You will evaluate fruity and ester aroma, floral aroma, grain aroma, cellar and fermentation aroma, sauce-like and roasted aroma, acidic aroma, alcoholic aroma, sweet aroma, overall aroma intensity, and aroma harmony. You will also judge the overall similarity between each reconstructed sample and its corresponding original Baijiu. All attributes will be rated on a 0-to-10 scale. A score of 0 indicates not perceived or completely different, whereas a score of 10 indicates extremely intense or highly similar. Assessors will first rate the aroma attributes independently and will then evaluate original-to-reconstructed similarity. Before formal evaluation, the research team will standardize the terminology and scale anchors. Each round is expected to last no more than 40 minutes, with additional rest whenever olfactory fatigue occurs.

4 Requirements before and during evaluation

- For 1 hour before evaluation, avoid alcohol, smoking, spicy or high-sugar foods, and strongly flavored foods or beverages.
- Do not wear perfume, fragranced products, or strongly scented skincare products on the day of evaluation.
- Make each judgment independently and do not discuss the samples with other assessors.
- Stop immediately and inform the research team if you experience nasal or throat irritation, headache, dizziness, nausea, breathing discomfort, or any other unusual symptom.

5 Potential risks and risk control

No sample consumption is required, and the overall risk is low. Because the original Baijiu has a high alcohol content and the reconstructed samples contain volatile flavor compounds, repeated short-term smelling may cause temporary olfactory fatigue, mild nasal or throat irritation, headache, dizziness, nausea, or aversion to a particular odor. The research team will use covered sensory glasses, limit individual exposure, maintain ventilation, and provide rest between samples and rounds. You may pause or stop the evaluation at any time. If significant discomfort occurs, the session will be stopped immediately and the research team will help you obtain appropriate medical support when needed.

6 Potential benefits

Participation is not expected to provide a direct health or economic benefit. Your evaluations will help determine whether the compounds assigned high contribution scores by the model explain the principal aroma profiles of different Baijiu styles. The results may support model refinement, mechanistic aroma research, theses, and scientific publications.

7 Costs, compensation, and research-related harm

There is no cost to participate. You will receive RMB 100 as compensation after completing the full evaluation. If you withdraw, this will not affect reasonable compensation for the work already completed. If a confirmed

research-related injury occurs while you are following the study procedures, the research team will assist with management, necessary medical support, and reasonable compensation in accordance with applicable Chinese law and the policies of Beijing Technology and Business University.

8 Voluntary participation and withdrawal

Participation is entirely voluntary. Refusing to participate or withdrawing will not affect your study, employment, assessment, relationship with teachers or supervisors, or any other lawful rights at Beijing Technology and Business University. You do not need to give a reason for withdrawal. Before the data are irreversibly anonymized and aggregated, you may request deletion of records that can still be linked to you. After aggregation and irreversible anonymization, the research team may no longer be able to identify and remove your individual data.

9 Privacy and data use

A participant code will be used instead of your name. Names and contact details will be stored separately from sensory scores and will be accessible only to authorized research staff and ethics reviewers when required. De-identified sensory data may be used for statistical analysis, model validation, theses, scientific publications, conference presentations, and future studies directly related to this project. No public report will contain information that can identify you. No biological samples, audio recordings, or video recordings will be collected. Research records will be stored securely in accordance with university research-record requirements, normally for at least 5 years after publication, and will then be destroyed or retained only in a non-identifiable form.

10 Ethical basis and contacts

The study will follow the principles of respect, voluntary participation, informed consent, risk minimization, and confidentiality. Selection and training of assessors will refer to ISO 8586:2023. Baijiu sensory procedures and terminology will refer to GB/T 33404-2016 and GB/T 33405-2016. The study also follows the informed-consent, withdrawal, and confidentiality principles of the 2024 revision of the World Medical Association Declaration of Helsinki. The study will be conducted only after ethics approval, and the approval number will be entered on the first page.

**Principal investigator**	________________________
**Telephone and email**	________________________
**Research ethics committee**	Scientific Research Ethics Committee, School of Food and Health, Beijing Technology and Business University
**Ethics contact telephone**	17692106402

11 Participant consent statement

☐ I have read and understood this document. The research team has explained the purpose, procedures, risks, and my rights in language that I understand.

☐ I have had the opportunity to ask questions and have received satisfactory answers.

☐ I understand that this is an orthonasal smelling study only and that I must not taste or swallow any sample.

☐ I understand that participation is voluntary and that I may withdraw at any time without disadvantage.

☐ I agree that my de-identified sensory scores may be used for statistical analysis, model validation, and research publication.

☐ I will receive a signed and dated copy of this consent form.

After receiving and considering the information above, I voluntarily agree to participate in this study.

12 Researcher declaration

I confirm that I have explained the purpose, procedures, possible risks and discomforts, privacy protections, and withdrawal rights to the participant. I have provided an opportunity for questions and have confirmed that the participant has made a voluntary decision without coercion or undue influence.

**Researcher name**	______________________________
**Researcher signature**	______________________________
**Date**	____ / ____ / ______
