# Supplementary material for: Construction of a Comprehensive Contribution Ranking Model for Baijiu Aroma Compounds Based on Multi-Source Molecular Feature Fusion and Validation by Aroma Recombination
Source: Foods. 2026 Aug 28;15(17):3043. doi: 10.3390/foods15173043 (PMC13565323; doi:10.3390/foods15173043)
Supplement: Supplementary file 1 [file foods-15-03043-s001.zip › Supplementary S5 Calculation Method and Parameter Description of the Comprehensive Contribution Ranking Model for Baijiu Aroma Compounds.pdf]

Calculation Method and Parameter Description of the Comprehensive Contribution Ranking Model for Baijiu Aroma Compounds

1. Reproducibility of Data Processing and Model Calculation

The computational workflow followed a fixed sequence. It included literature collection, database construction, chemical identity reconciliation, molecular structure standardization, descriptor calculation, module construction, sample specific scoring, and within sample ranking. The literature search covered the Web of Science Core Collection, Scopus, PubMed, and CNKI from January 2002 to December 2025. Reference lists of eligible studies were also examined. A total of 435 peer reviewed sources were retained. Compound identities, molecular properties, odor descriptors, sensory thresholds, occurrence evidence, and source information were entered into a structured master table. Supplementary S3 provides the complete list of literature sources. Supplementary S4 provides the compound records, field definitions, evidence status, and quality control results. The database was frozen before model calculation. No newly retrieved record was introduced during sample scoring or sensory recombination.

The released database contained 5,572 compound records. English names and CAS numbers were retained for reporting and manual verification. They were not used as the sole model identifiers. InChIKey was used as the unique structural key. Each SMILES string was parsed with RDKit and converted into a canonical isomeric SMILES representation. An InChIKey was regenerated from the parsed structure and compared with the recorded InChIKey. PubChem CID served as an additional identity field. The released table therefore contained 1 record for each unique InChIKey. Records with unresolved salt or mixture composition, conflicting PubChem structures, or insufficient structural certainty were marked through explicit quality control fields. A total of 4 unresolved records were excluded from model profile construction. The remaining 5,568 compounds entered the computable scorer. Their inclusion status and the reasons for exclusion are reported in Supplementary S4.

Missing information was handled by predefined rules. Blank cells in the compound table indicate unavailable, inapplicable, or unverified information unless a

corresponding status field gives a more specific interpretation. Missing values were not replaced by the mean or median of other compounds. A blank external odor evidence count was treated as 0 only when calculating the evidence module. If an odor threshold was unavailable, a fixed threshold prior of 1.0 mg/L was used in the concentration activation calculation. The same prior was applied to every affected compound. This prevented compounds with missing thresholds from receiving compound specific assumptions that could alter their relative order. A compound without a quantitative value because it was not detected in a sample was assigned a concentration of 0 for that sample. Its concentration activation was therefore 0. Any concentration supplied to the scorer had to be a finite and nonnegative value expressed in mg/L. Records without a resolvable identity or a computable molecular structure were not imputed and were excluded from scoring.

Molecular descriptors were calculated from the standardized structure. The descriptor values were transformed to the interval from 0 to 1 using the scaling rules reported in Supplementary S4 and the equations given below. The odor, physicochemical, molecular structure, matrix related, and evidence modules were also constrained to this interval before integration. Module weights were calculated by the stated softmax equation. All 5 module logits in the released model were fixed at 0. The resulting weight was 0.2 for each module. These weights were not refitted for individual compounds, samples, or Baijiu aroma styles. The current release also assigned the compound interaction term a value of 0 because validated pairwise adjacency effects were not available. Concentration activation, matrix correction, background adjustment, and score normalization were applied in the order defined by the equations below. The final percentage was calculated as $P_i=100S_i/\sum_j S_j$. The P_i values therefore sum to 100 within each sample. No score was manually adjusted after calculation.

The same frozen rules were used to construct the 23 sample level features described below. Feature selection and parameter determination were completed using the training and validation data. The independent test data were used only for final performance evaluation. Supplementary S12 contains the complete computational package. The file scorer.json stores the 5,568 compound profiles, module logits,

missing threshold prior, and sample level model coefficients. The file formula_registry.json records the mathematical definitions and calculation order. The file model_card.json records the input contract, model scope, random seed, and input file hashes. The file metrics.json provides the validation results. Source code, environment requirements, an example input file, and SHA256 checksums are also supplied. The recorded random seed was 20260714. Use of the same input, software version, and released model files reproduces the reported ranking and contribution percentages within ordinary floating-point precision.

2. Comprehensive Contribution Scoring of Compounds

For compound i, five normalized modules were constructed for odor, physicochemical properties, molecular structure, matrix-related properties, and evidence quality. The comprehensive contribution score is defined as a within-sample relative ranking index obtained by integrating these modules with concentration and matrix information for a specified Baijiu sample under the established model conditions. It does not represent the absolute sensory contribution of a compound. Each module value was constrained to the interval from 0 to 1. The modules were calculated as follows.

$$z_{i1} = \min\left(1, \max\left(0, \frac{I_i}{100}\right)\right)$$

$$z_{i2} = \frac{\left[\min\left(1, \max\left(0, \frac{MW_i}{500}\right)\right) + \min\left(1, \max\left(0, \frac{|LogP_i|}{8}\right)\right) + \min\left(1, \max\left(0, \frac{TPSA_i}{150}\right)\right)\right]}{3}$$

$$z_{i3} = \frac{\left[\min\left(1, \max\left(0, \frac{RC_i}{6}\right)\right) + \min\left(1, \max\left(0, \frac{ARC_i}{4}\right)\right) + \min(1, \max(0, FCSP3_i))\right]}{3}$$

$$z_{i4} = \frac{\left[\min\left(1, \max\left(0, \frac{HBA_i}{10}\right)\right) + \min\left(1, \max\left(0, \frac{HBD_i}{5}\right)\right) + \min\left(1, \max\left(0, \frac{RB_i}{15}\right)\right)\right]}{3}$$

$$z_{i5} = \min\left(1, \max\left(0, \frac{N_{s,i} + N_{e,i}}{8}\right)\right)$$

where z_{i1} to z_{i5} denote the odor, physicochemical-property, molecular-structure, matrix-related-property, and evidence-quality modules, respectively. I_i denotes Importance_Score_100. MW_i, $LogP_i$, and $TPSA_i$ denote molecular weight, the octanol-water partition coefficient, and topological polar surface area, respectively. RC_i, ARC_i, and $FCSP3_i$ denote ring count, aromatic ring count, and

the fraction of sp3-hybridized carbon atoms, respectively. HBA_i, HBD_i, and RB_i denote the numbers of hydrogen-bond acceptors, hydrogen-bond donors, and rotatable bonds, respectively. $N_{s,i}$ and $N_{e,i}$ denote the numbers of sensory odor-evidence sources and external odor sources, respectively.

Compound concentrations were standardized to mg/L. Let C_i be the mass concentration of compound i and T_i be its odor threshold. The odor activity value and concentration activation were calculated as follows.

$$OAV_i = \frac{C_i}{T_i}$$

$$a_i = \frac{\ln(1 + OAV_i)}{[1 + \ln(1 + OAV_i)]}$$

where OAV_i is the concentration-to-threshold ratio and a_i is the concentration activation compressed to the interval from 0 to 1. A missing threshold was assigned a prior value of 1.0 mg/L.

Let θ_m be the transformation parameter for module m and M be the set of five modules. Module weights were calculated using the softmax function.

$$w_m = \frac{\exp(\theta_m)}{[\sum_{(r \in M) \exp} \quad (\theta_r)]}$$

where w_m is the weight of module m. All five θ_m values in the released model were set to 0. Therefore, each module had a weight of 0.2.

Interactions between compounds were first subjected to soft-threshold shrinkage. Let q_i be the sum of the interaction effects connecting compound i to the other compounds in the sample. Then

$$\varphi_i = clip[sign(q_i) \max(|q_i| - 0.05, 0), -0.25, 0.25]$$

where φ_i is the sparsified interaction adjustment and clip denotes interval truncation. No interaction-adjacency effects were defined in the current release artifact. Therefore, φ_i was 0 in the actual calculations. The intrinsic potential H_i of a compound was calculated as

$$H_i = softplus\left\{\max\left[0, \sum_{(m \in M) w_m} z_{im} + \varphi_i\right]\right\}$$

where softplus(x) = max(x, 0) + ln[1 + exp(−|x|)] was used to ensure nonnegative scores.

Alcohol content, pH, aging time, and the aroma-type prior jointly defined the matrix adjustment. The matrix index e_i and adjustment factor g_i were calculated as follows.

$$e_i = \frac{\beta_{(iE)(E-52)}}{10} + \beta_{(ipH)(pH-3.5)} + \frac{\beta_{(iA)A}}{15} + b_{is} + \ln(\pi_{is})$$

$$g_i = \exp[clip(e_i, -8, 8)]$$

where E is the alcohol content and A is the aging time. $\beta_i E$, $\beta_i pH$, and $\beta_i A$ are the sensitivity coefficients for alcohol content, pH, and aging time, respectively. b_{is} is the bias associated with aroma type s, and π_{is} is the aroma-type prior adjustment coefficient. The pathway score r_i of a compound was calculated as

$$r_i = \max(0, H_i a_i g_i + \varphi_i)$$

Assume that a sample contains N compounds, which are ranked in descending order by r_i. The number of candidate compounds K and the background aggregate B of the noncandidate compounds were calculated as follows.

$$K = \min[500, \lceil 0.2N \rceil]$$

$$B = \left(\frac{1}{N-K}\right) \sum_{(j \notin TopK) r_j}$$

B was set to 0 when no noncandidate compounds were present. Let ρ_i be the background-affinity coefficient and $\mathbb{1}$ be the indicator function. The final compound score S_i and within-sample contribution P_i were calculated as follows.

$$S_i = softplus\{\max[0, r_i + \mathbb{1}_{(i \in TopK)\max}(0, \rho_i)\, B]\}$$

$$P_i = \frac{100 S_i}{\sum_{j=1}^{N} S_j}$$

P_i is expressed as a percentage, and the P_i values of all compounds within the same sample sum to 100%. Compounds were ranked in descending order of S_i or P_i.

3. Sample-Level Feature Construction and Parameter Determination

The sample-level model converted variable numbers of compound records into a fixed 23-dimensional feature vector. Let a sample contain n compounds, let z_{im} be the normalized value of compound i in module m, and let a_i be its concentration activation. The module mean μ_m was calculated as

$$\mu_m = \left(\frac{1}{n}\right) \sum_{i=1}^{n} a_i z_{im}$$

The five μ_m values correspond to odor_mean, physchem_mean, structure_mean, matrix_mean, and evidence_mean. Module dispersion was calculated as max(μ_m) minus min(μ_m). The values of $a_i z_{i1}$ were sorted in ascending order as $o_{(1)}$ to $o_{(n)}$. With h = max[1, floor(n/2)], the background odor feature was calculated as

$$odor_{background} = \left(\frac{1}{n}\right)\sum_{i=1}^{h} o_{(i)}$$

The mean, population standard deviation, and maximum were calculated after natural-log transformation of the concentrations. Let L_i be the log-transformed concentration of compound i. Then

$$L_i = \ln[1 + \max(0, C_i)]$$

$$\bar{L} = \left(\frac{1}{n}\right)\sum_{i=1}^{n} L_i$$

$$\sigma_L = \sqrt{\left[\left(\frac{1}{n}\right)\sum_{i=1}^{n(L_i-\bar{L})^2}\right]}$$

$$L\_(max) = \max\_(1 \leq i \leq n)(L\_i)$$

Let d_i be a normalized molecular descriptor of compound i. Its log-concentration-weighted value $\bar{d}$ was calculated as

$$\bar{d} = \frac{[\sum_{i=1}^{n} \max(L_i, 10^{-12})\, d_i]}{[\sum_{i=1}^{n} \max(L_i, 10^{-12})]}$$

The weighted descriptors comprised MW, LogP, TPSA, HBA, HBD, RotBonds, RingCount, AromaticRingCount, and FractionCSP3. The aging features were A/15 and sin(πA/15), and the module-interaction features were $\mu_1\mu_2$ and $\mu_3\mu_4$. Together, these features formed the 23-dimensional vector x. In model-input order, x_1 to x_5 were the five module means, x_6 was module dispersion, x_7 was the background odor feature, x_8 was A/15, x_9 and x_{10} were the two module-interaction terms, and x_{11} was sin(πA/15). x_{12} to x_{14} were $\bar{L}$, σL, and Lmax, respectively. x_{15} to x_{23} were the nine weighted molecular descriptors in the order listed above.

A synthetic sensory proxy was constructed from chemical features, perturbations at the parent-formulation-family level, and sample-level perturbations. Let u_f and u_s be mutually independent variables that follow the standard normal distribution. The proxy target y was defined as

$$y = softplus(\eta + 0.035u_f + 0.025u_s)$$

$$\eta = 1.25x_1 + 0.72x_2 + 0.48x_3 + 0.55x_4 + 0.82x_5 + 0.30x_6 + 0.26x_7 + 0.20x_8$$
$$+0.34x_9 - 0.18x_{10} + 0.10x_{11} + 1.74x_{12} + 1.00x_{13} + 0.53x_{14} + 0.17x_{15} - 0.14x_{16}$$
$$+0.11x_{17} + 0.08x_{18} - 0.05x_{19} + 0.07x_{20} + 0.12x_{21} - 0.06x_{22} + 0.09x_{23}$$

where η is the chemical signal formed by the 23 observable features, u_f is the random effect

for the parent formulation family, and u_s is the sample-level random effect. The random effects were not included in the prediction model.

Since η was calculated directly from x_1 to x_{23}, the synthetic proxy and the predictor vector were structurally related by design. This relationship was used to create a controlled recovery benchmark. It should not be interpreted as independence between the predictors and the target. No sensory observations from the experimental Baijiu samples were used to define or adjust the proxy equation. No target derived variable was returned to the predictor matrix. The family effect and sample effect remained hidden during fitting. Dataset integrity was examined using both sample_id and parent_recipe_id. After the explanatory worksheet rows were excluded, Supplementary S6, S8, and S7 contained 74980 training records, 10008 validation records, and 15012 test records, respectively. No overlap in valid sample identifiers or parent formulation identifiers was found among the 3 partitions. Scaling was learned only from the training records. Model selection used only the validation records. Test records did not participate in the released tuning procedure. The formal evaluation was restricted to the designated 9000 record subset in Supplementary S9. Consequently, the evaluation measures generalization of the specified synthetic relationship to unseen parent formulation families. It does not establish prediction of independent sensory responses in authentic Baijiu.

Sample-level prediction was performed using a ridge-regression candidate framework. Features were standardized using the training-set means and population standard deviations. The intercept was not penalized. Let β_0 be the intercept, β be the regression-coefficient vector, and α be the regularization strength. The parameters were estimated by minimizing

$$\hat{\beta} = \underset{\beta_0,\beta}{argmin} \left\{ \sum_{i=1}^{n} \left[y_i - \beta_0 - x_i^T \beta \right]^2 + \alpha \sum_{j=1}^{p} \beta_j^2 \right\}$$

The candidate feature counts were 8, 11, 14, 20, and 23, and α was evaluated at 0, 0.01, 0.1, 1, and 10. Parameters were selected by minimizing the validation-set RMSE, and the test set was not used for tuning. The final model retained all 23 features and used $\alpha = 0$. It was refitted on the combined training and validation sets. Under this setting, the regularization term was 0 and the final prediction head was equivalent to ordinary least squares. After transformation back to the original feature scale, the prediction equation was

$$\hat{y} = -1.116811 + 2.918119x_1 + 3.481303x_2 + 1.880351x_3 + 1.133671x_4 + 0.858257x_5 + 0.583725x_6 - 0.628286x_7 + 0.191712x_8$$

$$-8.942602x_9 + 3.858959x_{10} + 0.099960x_{11} + 1.717379x_{12} + 0.979233x_{13} + 0.533813x_{14} - 0.583268x_{15} + 1.423773x_{16}$$

$$+1.545256x_{17} - 0.124689x_{18} - 0.746041x_{19} - 0.101123x_{20} - 0.237564x_{21} - 0.088019x_{22} - 0.386130x_{23}$$

where ŷ is the predicted integrated intensity of the sample. The definitions and order of x_1 to x_{23} are identical to those in the feature vector described above.

4. Model Performance Analysis

Model performance was evaluated using the coefficient of determination (R^2), root mean square error (RMSE), and mean absolute error (MAE), which were calculated as follows.

$$R^2 = 1 - \frac{\sum_{i=1}^{n}(y_i-\hat{y}_i)^2}{\sum_{i=1}^{n}(y_i-\bar{y})^2}$$

$$RMSE = \sqrt{\left[\left(\frac{1}{n}\right)\sum_{i=1}^{n(y_i-\hat{y}_i)^2}\right]}$$

$$MAE = \left(\frac{1}{n}\right)\sum_{i=1}^{n|y_i-\hat{y}_i|}$$

where y_i is the synthetic sensory proxy value of sample i, $\hat{y}_i$ is the model-predicted value, $\bar{y}$ is the mean of all observed values, and n is the number of samples.

For the training set, R^2 was 0.978390, RMSE was 0.044038, and MAE was 0.035195. For the validation set, R^2 was 0.904337, RMSE was 0.049342, and MAE was 0.040491. For the 9,000 records in the formal independent test set, R^2 was 0.952018, RMSE was 0.045684, and MAE was 0.036464. The mean and standard deviation of the formal test residuals were −0.003935 and 0.045514, respectively. These results indicate that the model fitted and predicted the synthetic sensory proxy values of independent parent formulation families well. These metrics evaluate the sample-level synthetic proxy target and should not be interpreted as measures of accuracy for actual sensory responses.
